# An Immunocompetent Hepatic Organoid Model Reveals Conserved Hepatic Immune Dysregulation During *Leishmania donovani* Infection

**DOI:** 10.3390/ijms27167178

**Published:** 2026-08-11

**Authors:** María-Cristina González-Montero, Miguel Criado, Celia Fernández-Rubio, Yolanda Pérez-Pertejo, Rosa M. Reguera, Rafael Balaña-Fouce, Carlos García-Estrada

**Affiliations:** 1 Departamento de Ciencias Biomédicas, Facultad de Veterinaria, Universidad de León, Campus de Vegazana s/n, 24007 León, Spain; magom@unileon.es (M.-C.G.-M.); cferrb@unileon.es (C.F.-R.); myperp@unileon.es (Y.P.-P.); rmregt@unileon.es (R.M.R.); rbalf@unileon.es (R.B.-F.); 2 Instituto de Ganadería de Montaña, CSIC-Universidad de León, Finca Marzanas s/n, Grulleros, 24346 León, Spain; mcrib@unileon.es; 3 Instituto de Biomedicina (IBIOMED), Universidad de León, Campus de Vegazana s/n, 24007 León, Spain; 4 IBIOLEÓN (Instituto de Investigación Biosanitaria de León), Hospital de León, Altos de Nava s/n, 24071 León, Spain

**Keywords:** visceral leishmaniasis, *Leishmania donovani*, hepatic organoids, macrophages, immune dysregulation, interferon signaling, host–pathogen interactions

## Abstract

Visceral leishmaniasis, caused by *Leishmania donovani*, is characterized by profound alterations in hepatic immune responses that promote parasite persistence. However, the lack of physiologically relevant in vitro systems limits investigation of tissue-level host–pathogen interactions. An immunocompetent three-dimensional hepatic organoid model incorporating bone marrow-derived macrophages was established to investigate liver-stage immune responses to *L. donovani* infection. Integrated cytokine profiling and transcriptomic analyses were benchmarked against infected mouse liver tissue. Macrophages acquired functional characteristics consistent with a Kupffer cell-like phenotype and contributed to immune homeostasis within the co-culture. Infection induced a conserved immune response characterized by inflammatory and chemotactic activation. This response was accompanied by coordinated suppression of antimicrobial pathways, including the IL-12–IFN-γ axis and interferon-stimulated genes. These immune alterations were largely shared between the organoid model and infected liver tissue, although systemic metabolic reprogramming and multicellular complexity were not fully recapitulated. Our findings identify a conserved pattern of hepatic immune dysregulation in which inflammatory activation is uncoupled from effective antimicrobial signaling, potentially facilitating parasite persistence. This immunocompetent hepatic organoid platform provides a physiologically relevant system for investigating host–pathogen interactions and evaluating host-directed therapeutic strategies for visceral leishmaniasis.

## 1. Introduction

The liver plays a pivotal role at the interface between metabolism and immunity, acting as a primary immunological barrier against blood-borne pathogens [1,2]. This dual function is sustained by a complex cellular ecosystem in which parenchymal cells coexist with diverse innate immune populations, including self-maintaining, non-migratory tissue-resident phagocytic Kupffer cells and infiltrating monocyte-derived macrophages, that collectively regulate immune surveillance, inflammation, and tissue repair [3,4]. These cells contribute to pathogen recognition, cytokine production, antigen presentation, and orchestration of downstream immune responses [4,5]. Their functional polarization plays a decisive role in determining whether hepatic inflammation is resolved or progresses toward chronic pathology, and this tightly regulated balance between immune activation and tolerance in the liver is directly challenged during infection by intracellular parasites such as *Leishmania* spp., which specifically target macrophage populations [6].

*Leishmania* spp. are protozoan trypanosomatid parasites transmitted by phlebotomine sandflies, and are responsible for leishmaniasis, one of the most prevalent zoonotic neglected tropical diseases, with clinical manifestations ranging from cutaneous and mucocutaneous to visceral disease [7,8]. *Leishmania donovani* and *Leishmania infantum* (known as *Leishmania chagasi* in the New World) are the causative agents of visceral leishmaniasis (VL), the most severe presentation of the disease, with an estimated 50,000–90,000 new cases occurring annually worldwide (only 25–45% reported to WHO) and a fatality rate exceeding 95% if left untreated [9]. VL is characterized by persistent irregular fever, hepatosplenomegaly, weight loss, pancytopenia, and hypergammaglobulinemia [7], and the liver constitutes a major site of parasite clearance and immune regulation, where granuloma formation and macrophage-mediated effector mechanisms are central to host defense [6,10]. However, *Leishmania* parasites actively subvert macrophage antimicrobial pathways and reprogram host transcriptional responses, modulating cytokine networks involving TNF-α, IFN-γ, IL-10, and inducible nitric oxide synthase (iNOS) pathways to promote intracellular survival and persistence [11,12,13].

Traditional animal models of VL, such as mice and hamsters, have been relevant in elucidating disease pathogenesis, allowing detailed characterization of parasite burden and immune responses in organs like the liver and spleen, including Th1/Th2 cytokine profiles and the expression of iNOS, IL-10, and IFN-γ in infected tissues [13,14,15]. However, these models have limitations due to interspecies differences in disease progression and immune response, limiting direct extrapolation to human VL [15,16]. In this context, three-dimensional (3D) hepatic organoid models have emerged as physiologically relevant in vitro systems. Hepatic organoids derived from adult stem or progenitor cells preserve hepatobiliary differentiation capacity and maintain functional responses to environmental cues, offering distinct advantages over conventional two-dimensional (2D) cultures [17,18]. These 3D systems provide experimental accessibility not achievable in vivo, while faithfully recapitulating key aspects of liver physiology and pathology [19,20,21]. Despite these advances, most hepatic organoid models still lack the cellular complexity required to reproduce tissue-level immune responses during infection. In particular, the contribution of macrophages to the hepatic response against *Leishmania* and the extent to which immunocompetent organoid systems recapitulate the transcriptional programs observed in vivo remain poorly understood. Recent advances have led to the development of increasingly sophisticated immunocompetent hepatic organoid models incorporating macrophages and other non-parenchymal cells to study liver disease and host immune responses [22,23,24,25,26]. Nevertheless, whether these systems faithfully reproduce the hepatic immune transcriptional programs elicited during *Leishmania* infection remains unknown.

Recently, our group developed mouse organoids to establish a model for *Leishmania* infection, enabling the study of host–pathogen interactions in a controlled in vitro setting [27]. Building on this platform, the present study investigates whether incorporation of macrophages improves the physiological relevance of the model and whether the resulting immunocompetent organoid–macrophage co-culture reproduces key hepatic immune responses and transcriptional programs observed in vivo during *L. donovani* infection.

## 2. Results

### 2.1. Development and Characterization of an Immunocompetent In Vitro Model of Mouse Hepatic Organoids Co-Cultured with Mouse Macrophages

**Figure 1:**
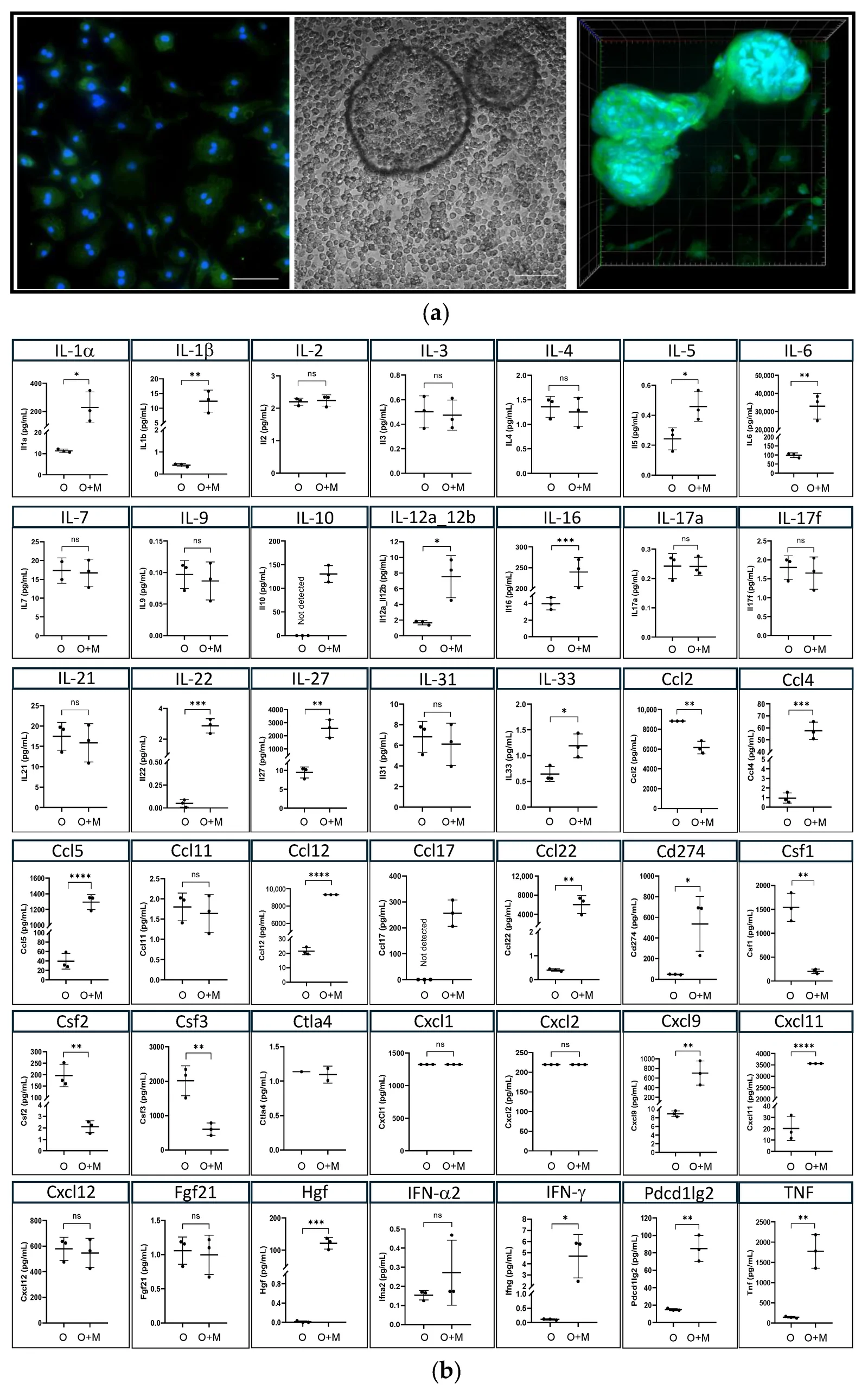
Co-culture of mouse hepatic organoids with bone marrow-derived macrophages and cytokine production: (**a**) representative images of: (left) bone marrow-derived macrophages after 24 h of culture (epifluorescence, scale bar = 50 µm); (middle) bone marrow-derived macrophages and mouse hepatic organoids after 24 h of co-culture (brightfield, scale bar = 100 µm); (right) 3D confocal reconstruction of bone marrow-derived macrophages and mouse hepatic organoids after 24 h of co-culture (epifluorescence; grid squares represent 30 × 30 µm). In the left and right micrographs, nuclei are stained with DAPI (blue). Both macrophages and hepatic organoids are labeled in green: macrophages with ionized calcium-binding adapter molecule 1 (Iba1) and organoids with Zonula Occludens 1 (ZO-1). Spatial separation of the two cell types allows their distinction despite sharing the same channel; (**b**) production of chemokines (pg/mL) by mouse hepatic organoids (O) and mouse hepatic organoids after 24 h of co-culture with murine bone marrow-derived macrophages (O+M). Results show the mean ± SD of three technical replicates determined using SigmaPlot^®^ version 10.0 (Grafiti LLC, Austin, TX, USA). ns, non-significant; * *p* ≤ 0.05; ** *p* ≤ 0.01; *** *p* ≤ 0.001; **** *p* ≤ 0.0001.

To generate an immunological component hepatic organoid model, mouse hepatic organoids previously developed by our group [27] were co-cultured with bone-marrow derived macrophages. In the absence of *L. donovani*, macrophages displayed an elongated adherent morphology, while a subset of cells were binucleated (Figure 1a, left panel). After 24 h of co-culture, hepatic organoids remained structurally intact and were closely associated with bone marrow-derived macrophages (Figure 1a, middle panel). Immunofluorescence images (Figure 1a, left and right panels) revealed that these macrophages expressed Iba-1, a well-established pan-macrophage marker used for the identification of Kupffer cells in the liver [28].

To confirm the immunocompetence of the system, cytokine levels were determined in the culture supernatants of organoids grown in the absence (O) and presence of macrophages (O+M) after 24 h co-culture (Figure 1b). The incorporation of macrophages into the murine hepatic organoid system resulted in a significant remodeling of the profile of secreted cytokines and chemokines, compared to organoids lacking immune cells. Specifically, macrophage-containing organoids displayed increased levels of the pro-inflammatory cytokines IL-1α, IL-1β, IL-6, and TNF, indicating functional activation of innate immune signaling pathways within the organoid. Concomitantly, elevated levels of immunoregulatory cytokines, including IL-10, IL-27, and IL-33, were observed. Increased production of IL-12 and IFN-γ, together with the IFN-γ-inducible chemokines CXCL9 and CXCL11, further indicates the ability of the model to support Th1-associated signaling and activate the IFN-γ–STAT1 axis under basal, non-infected conditions. The presence of macrophages also resulted in enhanced secretion of chemokines involved in immune cell recruitment and spatial organization, including CCL4, CCL5, CCL12, CCL17, and CCL22; whereas CCL2 levels were reduced. In addition, macrophage-containing organoids exhibited increased levels of hepatoprotective and regenerative factors such as HGF and IL-22, as well as elevated expression of immune checkpoint molecules CD274 (PD-L1) and PDCD1LG2 (PD-L2). In contrast, hematopoietic growth factors involved in myeloid cell expansion (CSF1, CSF2, and CSF3) decreased. Non-significant changes were detected in cytokines primarily associated with adaptive immune responses (IL-2, IL-4, IL-17A/F) or neutrophil-associated chemokines (CXCL1, CXCL2) (Figure 1b).

### 2.2. Transcriptional Response of the Immunocompetent In Vitro Model of Mouse Hepatic Organoids to L. donovani Infection

**Figure 2:**
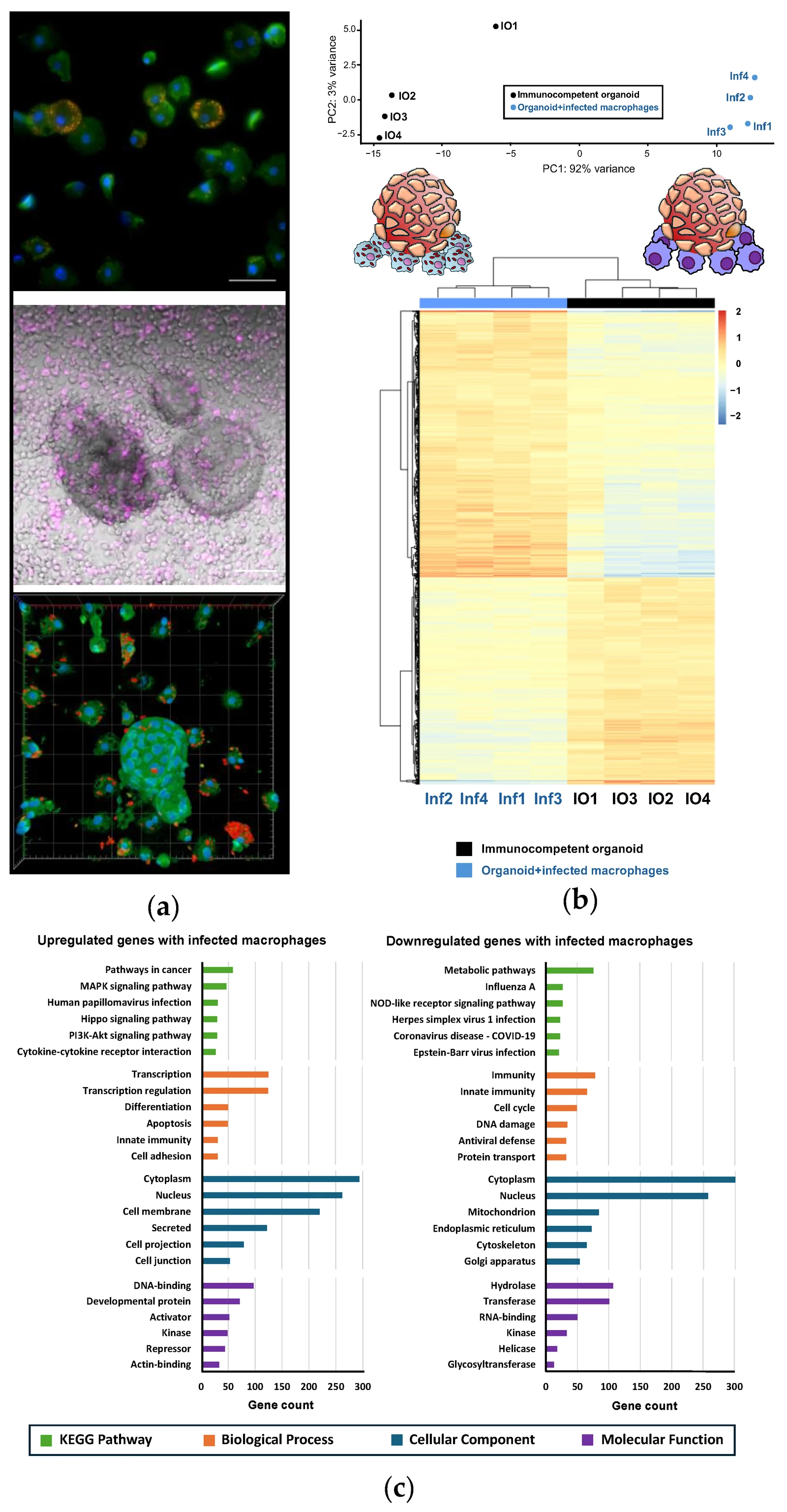
Global transcriptomic analysis of mouse hepatic organoids co-cultured with *L. donovani*-mCh-infected and non-infected macrophages: (**a**) representative micrographs of *L. donovani* infection in bone marrow-derived macrophages and hepatic organoid co-cultures; (top) bone marrow-derived macrophages infected with *L. donovani*-mCh amastigotes after 24 h of culture (epifluorescence; scale bar = 50 µm); (middle) infected bone marrow-derived macrophages co-cultured with mouse hepatic organoids for 24 h, shown as a merged brightfield and epifluorescence overlay (scale bar = 100 µm); (bottom) 3D confocal reconstruction of the co-culture of bone marrow-derived macrophages infected with *L. donovani*-mCh amastigotes and hepatic organoids at 24 h (epifluorescence; grid squares represent 30 × 30 µm). In the top and bottom micrographs, nuclei are counterstained with DAPI (blue). Macrophages and organoids are both immunolabeled in green (Iba1 and ZO-1, respectively); their distinct spatial distribution allows for clear visual separation despite sharing the same fluorescence channel. The fluorescence in the top micrograph is displayed in true color, acquired via a polychromatic camera under simultaneous excitation (*L. donovani*-mCh signal in orange color). For the monochrome-acquired images, the *L. donovani*-mCh signal is pseudocolored in magenta in the brightfield overlay (middle micrograph) to enhance contrast and ensure colorblind accessibility, and in red in the confocal reconstruction (bottom); (**b**) transcriptional analysis of the immunocompetent mouse hepatic organoids and mouse hepatic organoids co-cultured with macrophages infected with *L. donovani*-mCh. The principal component analysis (upper panel) was performed with samples of immunocompetent mouse hepatic organoids (IO1–IO4, black dots) and mouse hepatic organoids co-cultured with macrophages infected with *L. donovani*-mCh (Inf1-Inf4, blue dots). The heatmap (lower panel) shows the genes with the highest differential expression between immunocompetent mouse hepatic organoids (IO1–IO4) and mouse hepatic organoids co-cultured with macrophages infected with *L. donovani*-mCh (Inf1–Inf4). The applied filters are: Log2FC < −1 or >1, and q-value < 0.1. Color scale indicates gene expression levels from low (blue) to high (red). Dendrograms reflect hierarchical clustering based on sample similarity; (**c**) functional enrichment analysis of the differentially expressed genes in mouse hepatic organoids co-cultured with macrophages infected with *L. donovani*-mCh. The left panel shows the analysis of the 1062 upregulated genes, while the right panel shows the analysis of the 829 downregulated genes. The six most represented pathways in each category (KEGG pathway, biological process, cellular compartment, and molecular function) are represented as colored bars. The analysis was carried out with the DAVID free software (v2024q4).

Mouse hepatic organoids were co-cultured for 24 h with bone marrow-derived macrophages infected with *L. donovani*-mCh amastigotes to establish an immunocompetent model of infection. Infected macrophages displayed a rounded morphology, with reduced adherence, and contained variable numbers of amastigotes (Figure 2a, upper panel). The fluorescence emitted by the mCherry protein served as a proxy for parasite viability. The co-culture of hepatic organoids with *L. donovani*-infected macrophages was visualized using a combination of epifluorescence and brightfield microscopy in both non-fixed and fixed samples (Figure 2a).

Differences in gene expression profiles between immunocompetent in vitro co-culture model and mouse hepatic organoids co-cultured with infected macrophages were determined by RNA-seq. Principal component analysis revealed well-defined clusters between both groups (Figure 2b, upper panel). Although replicate 1 (IO1) of the immunocompetent co-culture model clusters slightly apart from the control group, it cannot be considered as an outlier. The clear separation primarily explained by Principal Component 1, which accounts for 92% of the total variance, highlights its key role in group discrimination (Figure 2b). Differential expression is represented in the heatmap (Figure 2b, lower panel), revealing that of the total 11,674 co-expressed genes in both conditions, 1062 genes (9.1%) were upregulated and 829 (7.1%) downregulated in the co-culture including *L. donovani*-mCh infected macrophages. A detailed characterization of the changes in the gene expression induced by the infection by *L. donovani*-mCh was performed by functional enrichment analysis of the differentially expressed genes (1062 upregulated and 829 downregulated). The top six most significant metabolic pathways, based on the KEGG results, as well as biological, cellular, and molecular categories, were identified for the upregulated and downregulated genes (Figure 2c). According to these results, the parasitic infection provoked high expression of genes associated with an inflammatory microenvironment reprograming cell signaling and promoting cell adaptation in the hepatic organoid, transcription and transcriptional regulation, differentiation and apoptosis. Cytoplasmic and nuclear compartments were identified as the primary sites of cellular activity, with an abundance of genes encoding DNA-binding proteins being transcribed. Conversely, *L. donovani*-mCh infection suppressed genes involved in metabolic functions, innate immunity, cell cycle, and protein transport. This downregulation was most prominent within these same compartments and particularly affected genes encoding hydrolases (Figure 2c).

The six most upregulated and downregulated genes in mouse hepatic organoids co-cultured with infected macrophages are included in Table 1 and Table 2, respectively.

Analysis of the top six most overexpressed genes (Table 1) revealed that they are predominantly associated with inflammatory processes, cellular stress responses, and the regulation of signaling pathways. In contrast, a significant repression of classical IFN-dependent response genes (*Ifit2*, *Ifit3* and *Ifi44*) was observed (Table 2).

To further characterize the effect of *L. donovani*-mCh on the host’s immune response, a detailed analysis of the differentially expressed genes involved in this process was conducted. From the 1062 upregulated genes, 94 genes were related to immunity-related processes (Supplementary Table S1). A robust upregulation of genes related to proinflammatory cytokine signaling, innate immune recognition, and antimicrobial defense was observed. Genes encoding proinflammatory cytokines (*Il1a*, *Il1b*, *Tnf*, and *Mif*) were upregulated. Increased expression was also observed for cytokine receptor genes (*Il22ra1*, *Il9r*, and *Il17rd*), chemokine genes (*Cxcl1*, *Cxcl2*, *Ccl3*, *Ccl4*, *Ccl8*), complement components (*C1qa*, *C1qb* and *C4b*), pattern-recognition receptors (*Cd14* and *Marco*), antimicrobial genes (*Lcn2*, *Duox2*, and *Slpi*), intracellular signaling molecules (*Map3k8*, *Card10* and *Rasgrp4*), negative regulators of inflammation (*Nfkbia*, *Tnfaip3*, and *Nr4a1/3*), transcription factors (*Runx1*, *Bach2*, and *Gata3*), macrophage-associated genes (*Ly6c2* and *Arg1*), neutrophil-associated genes (*Ly6g2* and *Retn*), and apoptosis- and stress-related genes (*Bik*, *Gadd45a/b/g*) (Supplementary Table S1). Conversely, from the 829 genes downregulated in organoids with *Leishmania*-infected macrophages compared to controls, 110 genes were found related to immunity (Supplementary Table S2). Among the downregulated genes cytokines and cytokine receptors (*Il12b* and *Il15ra*), IFN-inducible genes (*Ifit1–3*, *Ifi44*, and *Oasl1/2*), pattern-recognition receptors (*Tlr4*, *Tlr7*, *Ddx58*, and *Mavs*), inflammasome-associated genes (*Casp1* and *Pycard*), apoptosis-related genes (*Bid* and *Bik*), macrophage-associated genes (members of the *Gbp* family and *Mrc1*), and transcription factors (*Batf2* and *Arid5a*), were found.

**Figure 3:**
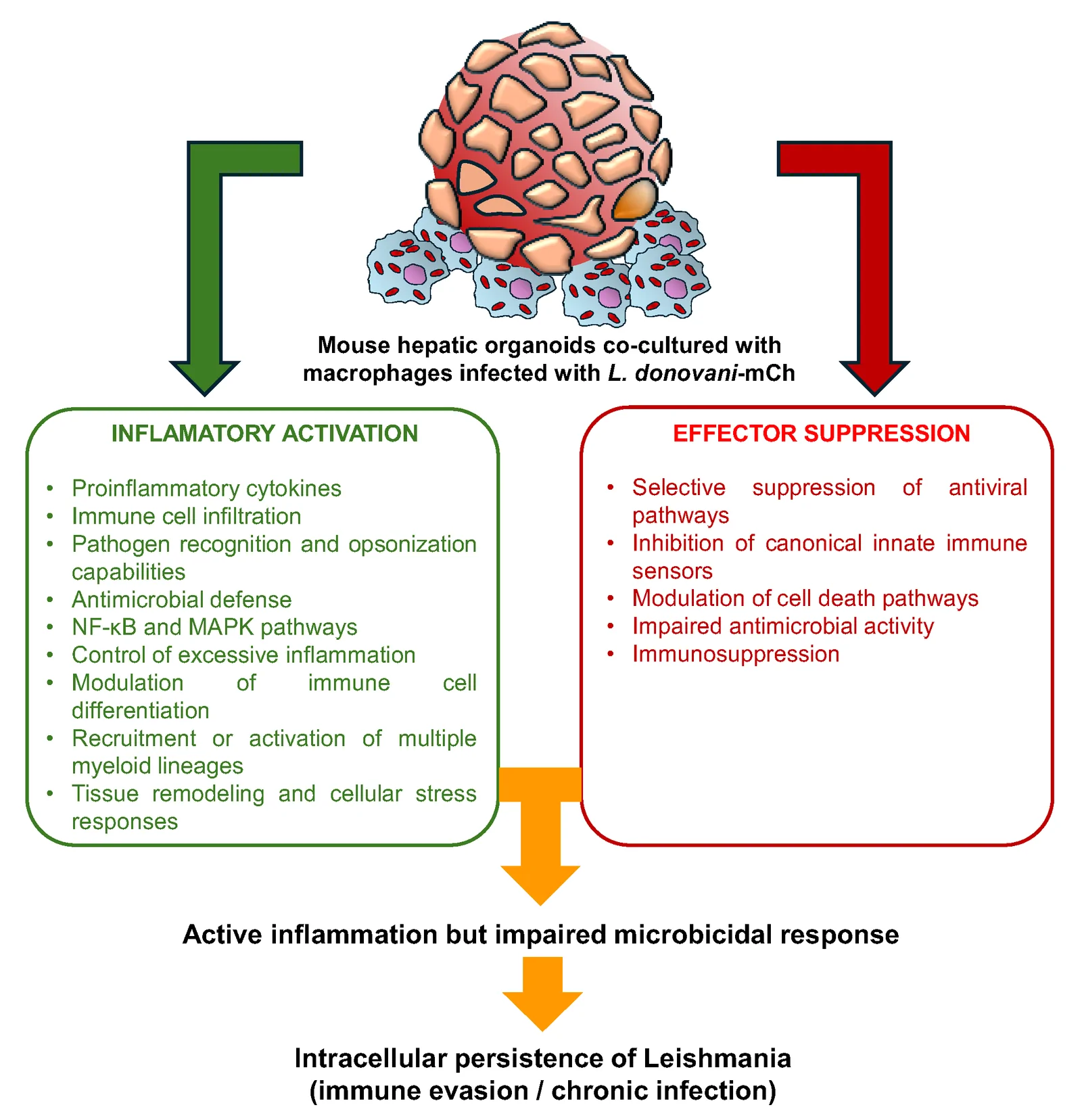
Integrated model of the host response to *L. donovani* infection based on RNA-seq data.

Overall, *L. donovani* infection induced extensive transcriptional remodeling in hepatic organoid–macrophage co-cultures, characterized by coordinated upregulation and downregulation of genes involved in immune and inflammatory processes. These data highlight the dual role of *Leishmania* in triggering host inflammatory defenses while simultaneously subverting antiviral and certain innate immune mechanisms, shaping a permissive environment for parasite persistence (Figure 3).

### 2.3. Leishmania-Infected Macrophages Shape the Immune Microenvironment of Mouse Hepatic Organoids

**Figure 4:**
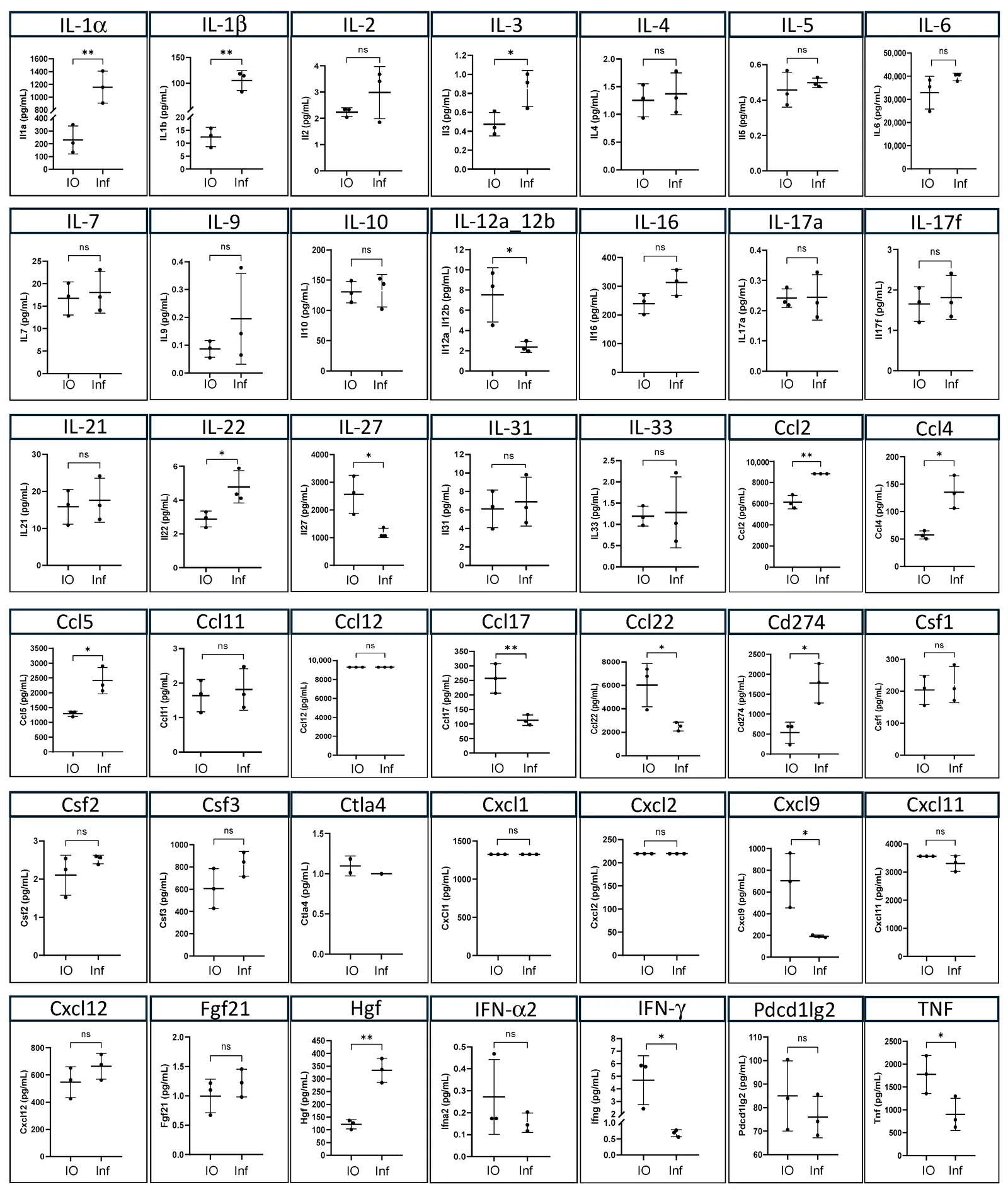
Effect of infected macrophages in the release of immunological mediators. Production of chemokines (pg/mL) by immunocompetent mouse hepatic organoids (IO) and mouse hepatic organoids after 24 h of co-culture with murine bone marrow-derived macrophages infected with L. donovani-mCh (Inf). Results show the mean ± SD of three technical replicates. ns, non-significant; * *p* ≤ 0.05; ** *p* ≤ 0.01.

In our previous work we observed that co-culture of *L. donovani* axenic amastigotes with mouse hepatic organoids led to a reduction in most of the cytokines produced by the organoid pointing to a marked decrease in Th1 and Th17 axes, as well as a reduction in cytokines that regulate lymphocyte and myeloid cell proliferation and activity [27]. To characterize the effect of infected macrophages in the release of immunological mediators in the 3D system, cytokine levels were determined in the culture supernatants of organoids co-cultured either with infected or uninfected macrophages (24 h co-culture) (Figure 4). Compared with organoids co-cultured with uninfected macrophages, infection induced increased secretion of IL-1α, IL-1β, IL-3, IL-22, CCL2, CCL4, CCL5, CD274 (PD-L1), and HGF. In contrast, IL12A, IL12B, IL-27, IFN-γ, TNF, CXCL9, CCL17, and CCL22 were significantly reduced. No significant differences were detected for IL-2, IL-4, IL-6, IL-10, IL-17A/F, CSF1, CSF2, CSF3, CXCL1, CXCL2, CXCL11, CXCL12, or PDCD1LG2 (Figure 4).

Overall, infection induced marked changes in the cytokine profile of macrophage-containing hepatic organoids with a selective and coordinated remodeling of inflammatory signaling rather than global immune activation.

### 2.4. Specific Role of Macrophages in the Hepatic Organoid During the L. donovani-mCh Infection

To define the contribution of macrophages to the transcriptional response of the organoid model, RNA-seq profiles from hepatic organoids co-cultured with *L. donovani*-mCh-infected macrophages were compared with those from organoids co-cultured with *L. donovani*-iRFP axenic amastigotes.

**Figure 5:**
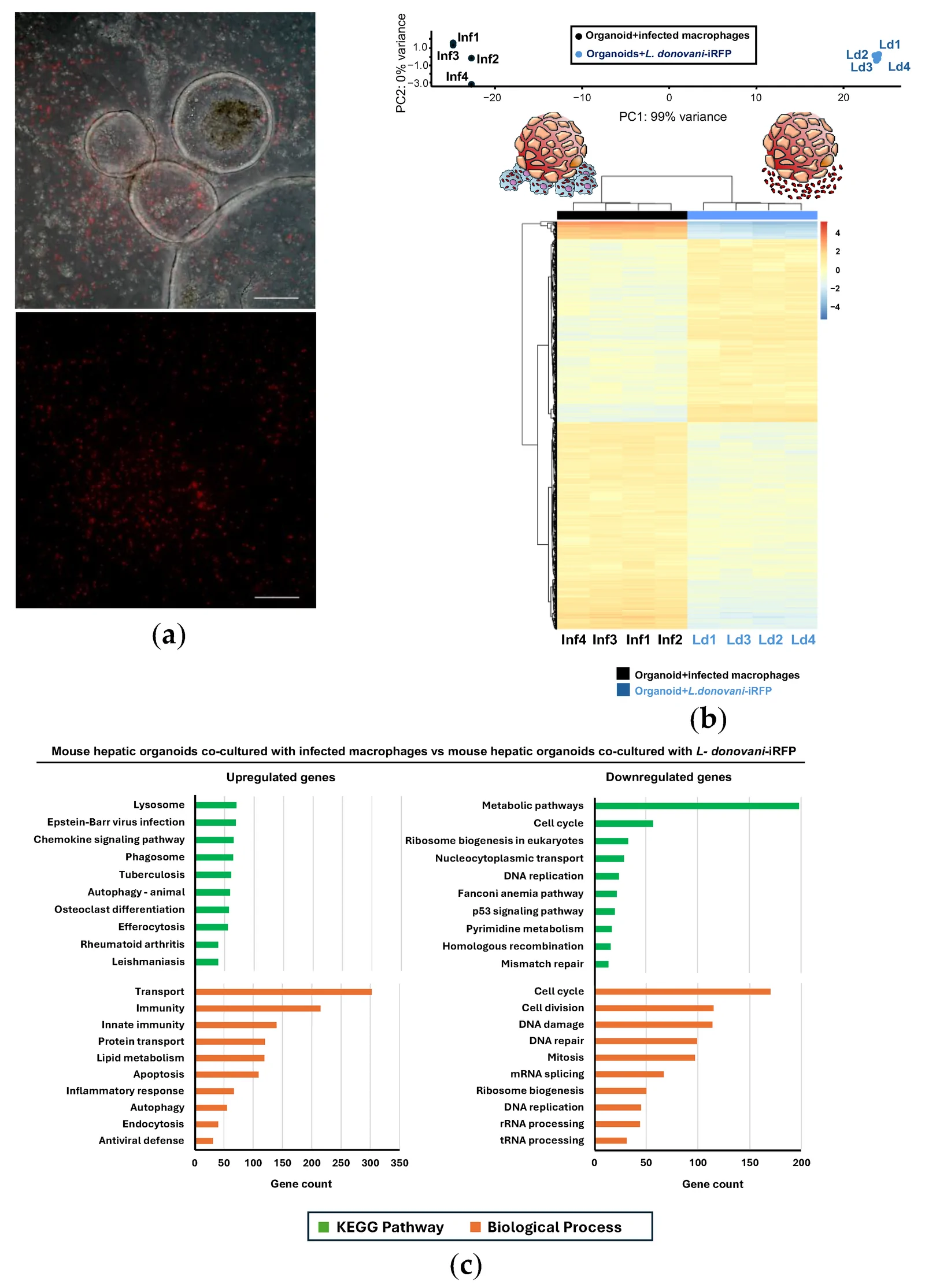
Global transcriptomic analysis of the role of macrophages in the hepatic organoid during *L. donovani* infection: (**a**) Co-culture of mouse hepatic organoids with *L. donovani*-iRFP amastigotes. Brightfield image shows organoid morphology, and iRFP signal (red) indicates the presence of viable amastigotes. Scale bar = 100 µm. (**b**) Transcriptional analysis of the mouse hepatic organoids co-cultured with macrophages infected with *L. donovani*-mCh and mouse hepatic organoids co-cultured with amastigotes of *L. donovani*-iRFP. Principal component analysis performed with samples of mouse hepatic organoids co-cultured with macrophages infected with *L. donovani*-mCh (Inf1-Inf4, black dots) and mouse hepatic organoids co-cultured with *L. donovani*-iRFP (Ld1–Ld4, blue dots) (upper panel). The heatmap (lower panel) shows the genes with the highest differential expression between macrophages infected with *L. donovani*-mCh (Inf1-Inf4) and mouse hepatic organoids co-cultured with *L. donovani*-iRFP (Ld1–Ld4). The applied filters are: Log2FC < −1 or >1, and q-value < 0.1. Color scale indicates gene expression levels from low (blue) to high (red). Dendrograms reflect hierarchical clustering based on sample similarity. (**c**) Functional enrichment analysis of the differentially expressed genes in mouse hepatic organoids co-cultured with *L. donovani*-iRFP amastigotes in comparison with mouse hepatic organoids co-cultured with infected macrophages. The left panel shows the analysis of the 2229 upregulated genes, while the right panel shows the analysis of the 2738 downregulated genes. The ten most represented pathways in the KEGG pathway and biological process are represented as colored bars. The analysis was carried out with the DAVID free software (v2024q4).

*L. donovani*-iRFP viability in the 3D system was confirmed by visualization of the fluorescence emitted by the iRFP produced by the amastigotes (Figure 5a). RNA-seq analysis was conducted, and principal component analysis revealed well-defined clusters between both groups, with no outliers, and a clear separation primarily explained by Principal Component 1, which accounts for 99% of the total variance (Figure 5b, upper panel). From the total of 11,511 co-expressed genes in both conditions, 2738 genes (23.8%) were upregulated and 2229 (19.4%) were downregulated in the co-culture of hepatic organoids with *L. donovani*-mCh-infected macrophages. Overexpressed and repressed genes are clearly visualized according to significant fold changes and statistical evidence in the heatmap (Figure 5b, lower panel).

For a detailed characterization of the role played by macrophages in the 3D in vitro model, functional enrichment analysis of the differentially expressed genes was carried out. The ten most significant metabolic pathways based on the KEGG results and the biological process were identified for each category (Figure 5c). Genes upregulated in hepatic organoids co-cultured with *L. donovani*-mCh-infected macrophages were strongly enriched in immune-related pathways, including KEGG categories such as Leishmaniasis, phagosome, lysosome, chemokine signaling pathway, autophagy, and tuberculosis, as well as biological processes associated with innate immunity, inflammatory response, endocytosis, apoptosis, and antimicrobial defense. Notably, the enrichment of the KEGG “Leishmaniasis” category contains 39 genes included within the group of upregulated genes after the co-culture of the organoids with *L. donovani*-mCh-infected macrophages (Supplementary Table S3). These genes are involved in Fc receptor-mediated phagocytosis, antigen presentation via MHC class II, inflammatory cytokine signaling, pathogen recognition, and IFN-γ-dependent antimicrobial responses.

In contrast, downregulated genes in the system including macrophages were predominantly associated with metabolic pathways, DNA replication and genome maintenance, including KEGG pathways such as mismatch repair, homologous recombination, Fanconi anemia, p53 signaling, and cell cycle, together with biological processes related to DNA repair, DNA damage response, mitosis, cell division, and ribosome biogenesis (Figure 5c).

The six most upregulated and downregulated genes when *L. donovani*-mCh-infected macrophages were co-cultured with mouse hepatic organoids in comparison with the 3D model in co-culture with *L. donovani*-iRFP axenic amastigotes are included in Table 3 and Table 4, respectively.

The overexpressed genes in Table 3 include antigen presentation genes (MHC I) and cathepsins. In this regard, cathepsin D (CtsD) in macrophages plays a central role in restorative functions and matrix degradation during the resolution of liver fibrosis [29], which suggests that the model including macrophages could impact immunomodulatory and tissue remodeling processes in the hepatic organoid. On the other hand, the repressed genes (Table 4) converge on proliferative activity (*Ccnd1* and *Ccnd2*), matrix remodeling (Timp3), pro-immune signaling (*Cxcl5*), and cellular stress response (*Ankrd1* and *Dkc1*).

**Figure 6:**
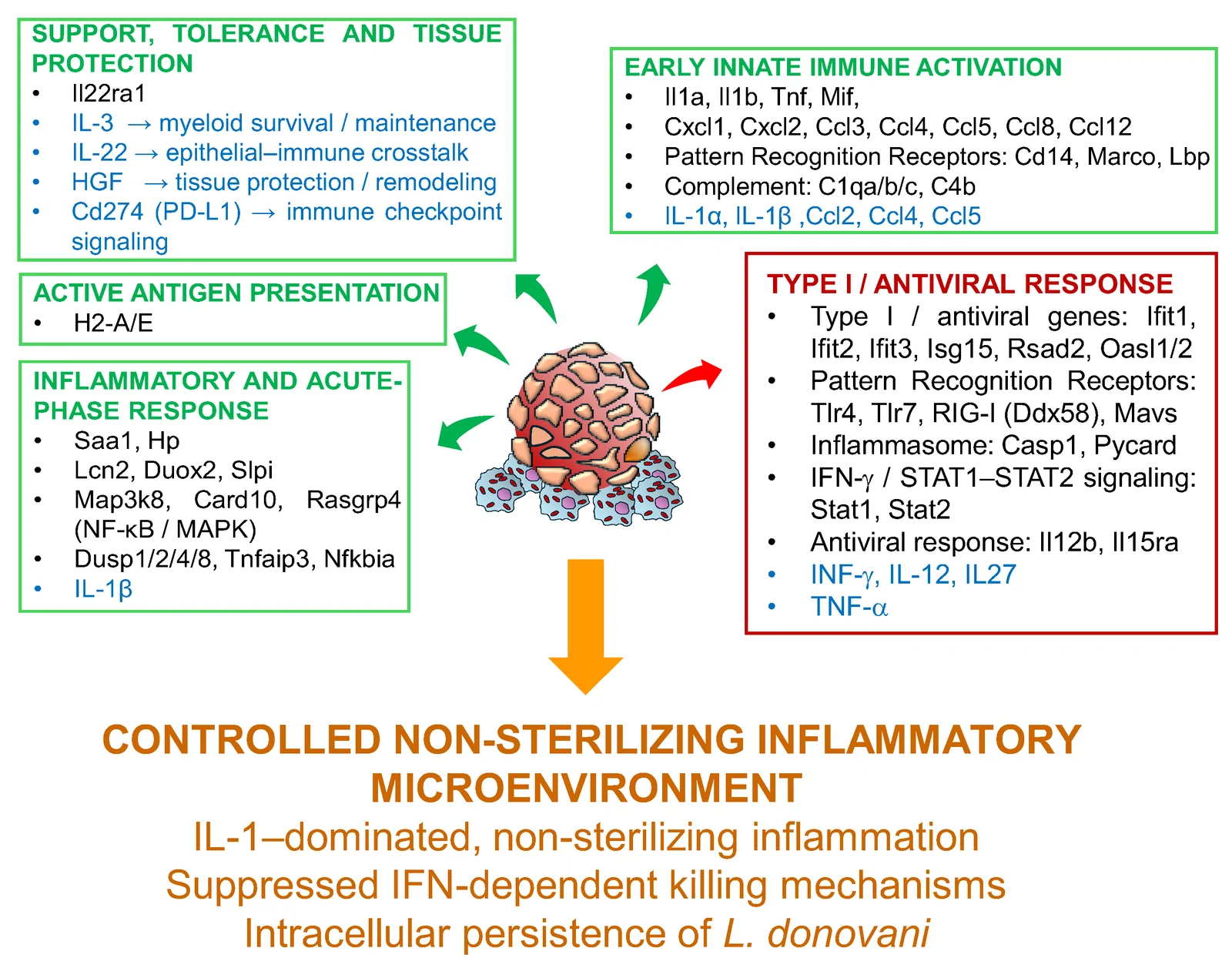
Role of macrophages in the hepatic organoid during *L. donovani* infection. *L. donovani*–infected macrophages reshape the immune microenvironment of murine hepatic organoids by inducing a strong IL-1-driven inflammatory response together with acute-phase and antimicrobial programs, while simultaneously activating tissue-protective and tolerogenic signals and selectively suppressing interferon-dependent and intracellular killing pathways. This coordinated remodeling results in a controlled but non-sterilizing inflammatory state that favors parasite persistence. Data from transcriptomics studies is indicated in black color, while data from cytokine analysis is indicated in blue color. Upregulated responses are indicated in green color, while downregulated responses are indicated in red color.

Overall, comparison with organoids exposed to axenic amastigotes showed that the presence of infected macrophages profoundly altered the transcriptional response of hepatic organoids, particularly in pathways related to immune function and host defense, simultaneously acting as defensive effectors and targets of subversion by *L. donovani* (Figure 6).

### 2.5. In Vivo Transcriptomic Profiling of Leishmania-Infected Mouse Liver Reveals Multicellular Host Responses Beyond Hepatocyte-Intrinsic Programs

**Figure 7:**
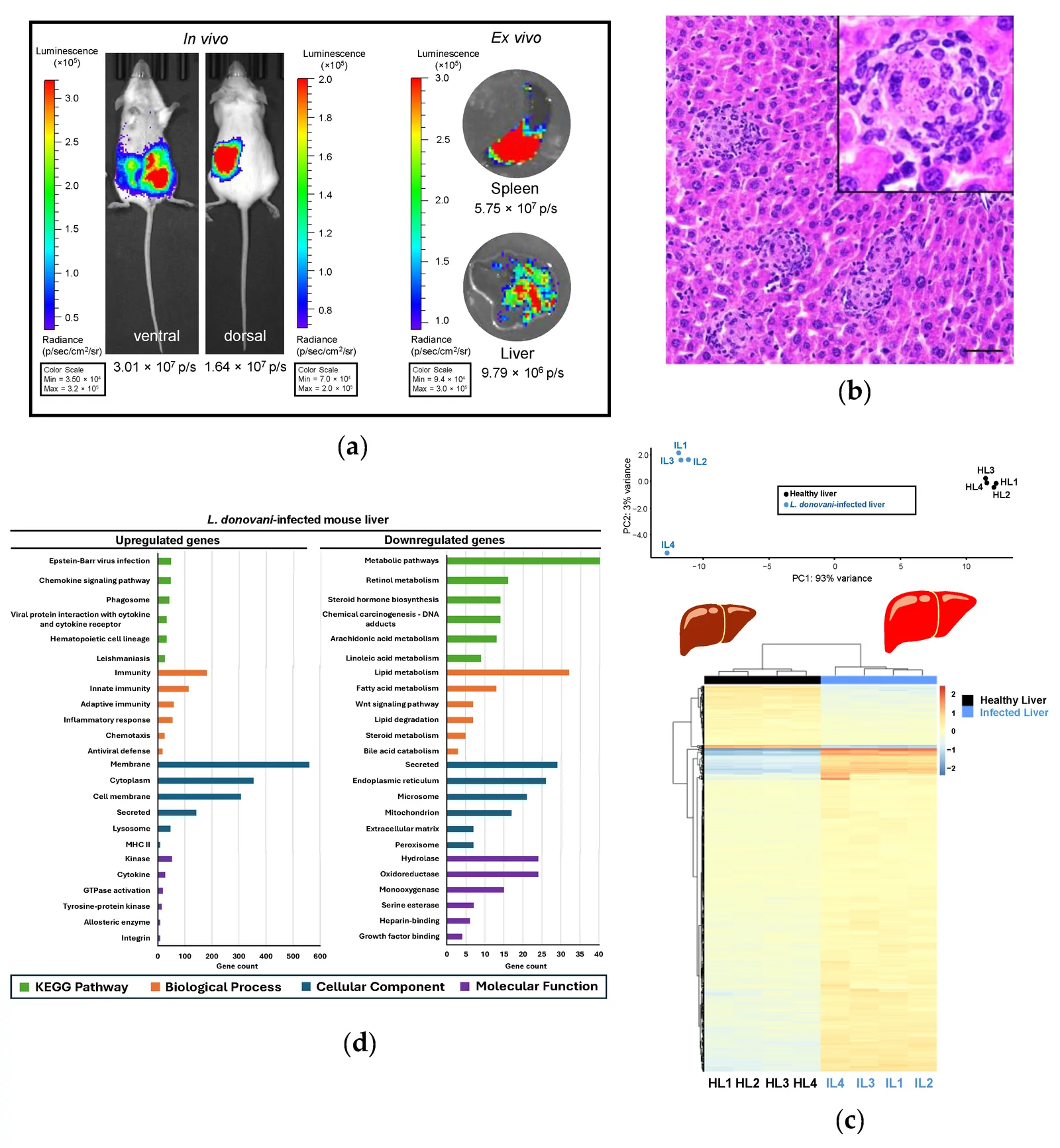
Global transcriptomic analysis of *Leishmania*-infected mouse liver: (**a**) In vivo and ex vivo imaging of *L. donovani*-PpyrE9h infection in a BALB/c mouse. Ventral and dorsal in vivo images of a BALB/c mouse infected with *L. donovani*-PpyRE9h taken 8 weeks post-infection and after intraperitoneal injection of D-luciferin. The liver and spleen of the humanely euthanized mouse were incubated with D-luciferin for ex vivo imaging analysis. Bioluminescence images were taken in an IVIS Spectrum (PerkinElmer, Shelton, CT, USA) and bioluminescence was expressed as total flux (photons/s) below each image. (**b**) Histological examination of the liver revealed a multifocal granulomatous hepatitis, characterized by uniformly distributed, well-defined granulomas measuring approximately 50–100 µm in diameter. Hematoxylin and eosin. Scale bar = 50 µm. (**c**) Transcriptional analysis of the liver from a healthy animal and the liver from a *L. donovani*-infected mouse. Principal component analysis performed with samples from healthy mouse livers (HL1-HL4, black dots) and *L. donovani*-PpyRE9h-infected mouse livers (IL1-IL4, blue dots) (upper panel). The heatmap (lower panel) shows the genes with the highest differential expression between healthy mouse livers (HL1-HL4) and mouse livers infected with *L. donovani*-PpyRE9h (IL1-IL4). The applied filters are: Log2FC < −1 or >1, and q-value < 0.1. Color scale indicates gene expression levels from low (blue) to high (red). Dendrograms reflect hierarchical clustering based on sample similarity. (**d**) Functional enrichment analysis of the differentially expressed genes in *L. donovani*-infected mouse livers in comparison with healthy livers. The left panel shows the analysis of the 1126 upregulated genes, while the right panel shows the analysis of the 218 downregulated genes. The six most represented pathways in each category (KEGG pathway, biological process, cellular compartment, and molecular function) are represented as colored bars. The DAVID free software (v2024q4) was used in the analysis.

To benchmark the organoid model against an in vivo response, transcriptomic profiles from the liver of a representative healthy mouse and a mouse infected for 8 weeks with *L. donovani*-PpyrE9h were compared. Following confirmation of infection in vivo and ex vivo (Figure 7a), the liver’s inflammatory status was confirmed by the presence of hepatic granulomas, which comprised a few large macrophages with abundant cytoplasm containing numerous intracytoplasmic basophilic structures, morphologically consistent with amastigotes, that were surrounded by a rim of mononuclear inflammatory cells, including macrophages and lymphocytes (Figure 7b). RNA-seq analysis was performed on four spatial replicates per liver. Principal component analysis revealed well-defined clusters between both groups, with a clear separation primarily explained by Principal Component 1, which accounts for 93% of the total variance, thus providing clear group discrimination (Figure 7c, upper panel). While the spatial replicates from the uninfected healthy liver (HL) clustered tightly, one spatial replicate from the infected liver (IL) (sample 4) showed slight divergence. This variance along the horizontal component reflects the expected intra-organ spatial heterogeneity typical of granulomatous inflammation.

Differential expression analysis identified 1126 upregulated genes (8.6%) and 218 downregulated genes (1.7%) among the 13,054 co-expressed genes in the infected liver compared to the healthy control. These overexpressed and repressed genes are clearly visualized according to significant fold changes and statistical evidence in the heatmap (Figure 7c, lower panel). To gain a more profound understanding of the *Leishmania* infection, functional enrichment analysis of the differentially expressed genes was performed. The top six most significant metabolic pathways, based on the KEGG results, as well as biological, cellular, and molecular categories, were identified for the upregulated and downregulated genes (Figure 7d).

The parasitic infection provoked high expression of genes associated with infection, chemokine signaling and immunity. The membrane and cytoplasm were identified as the most active cellular sites, while genes encoding kinases, cytokines and proteins involved in chemokine signaling were abundantly transcribed. Supplementary Table S4 includes those genes upregulated in *L. donovani*-infected liver that encode proteins related to chemokine signaling and cytokines. Interestingly, KEGG pathway enrichment analysis identified “Leishmaniasis” category as a significantly enriched pathway among upregulated hepatic genes. This enrichment was driven by 27 genes involved in Fc receptor-mediated phagocytosis, antigen presentation via MHC class II, inflammatory cytokine signaling, pathogen recognition, and IFN-γ-dependent antimicrobial responses (Supplementary Table S5). On the other hand, in the infected liver, a repression in the genes related to different metabolic pathways, including lipid and fatty acid metabolism, was observed. The secreted proteins and endoplasmic reticulum were identified as the main cellular compartments where most gene products were downregulated, and genes encoding hydrolases and oxidoreductases were mostly downregulated (Figure 7d).

The six most upregulated and downregulated genes in the liver infected with *L. donovani*-PpyrE9h are included in Table 5 and Table 6, respectively.

In the liver of the infected animal, several genes involved in immune activation and antigen presentation were upregulated (Table 5), the majority being associated with major histocompatibility complex class II (MHC II) function. In addition, *Iigp1*, a gene induced by IFN signaling and linked to host defense responses, showed increased expression in the infected liver. In contrast, a set of genes was downregulated in the infected mouse compared with the healthy control. These included *Pzp* and *Hamp2*, the latter involved in hepatic homeostasis, as well as several genes related to xenobiotic and lipid metabolism, such as *Cyp2c69*, *Cyp2a22*, *Cyp4a14*, and *Acot3* (Table 6).

Overall, infection was associated with extensive transcriptional remodeling, characterized by enrichment of immune-related pathways among upregulated genes and metabolic pathways among downregulated genes.

### 2.6. In Vivo Versus in Vitro Models of Hepatic Leishmania Infection

**Figure 8:**
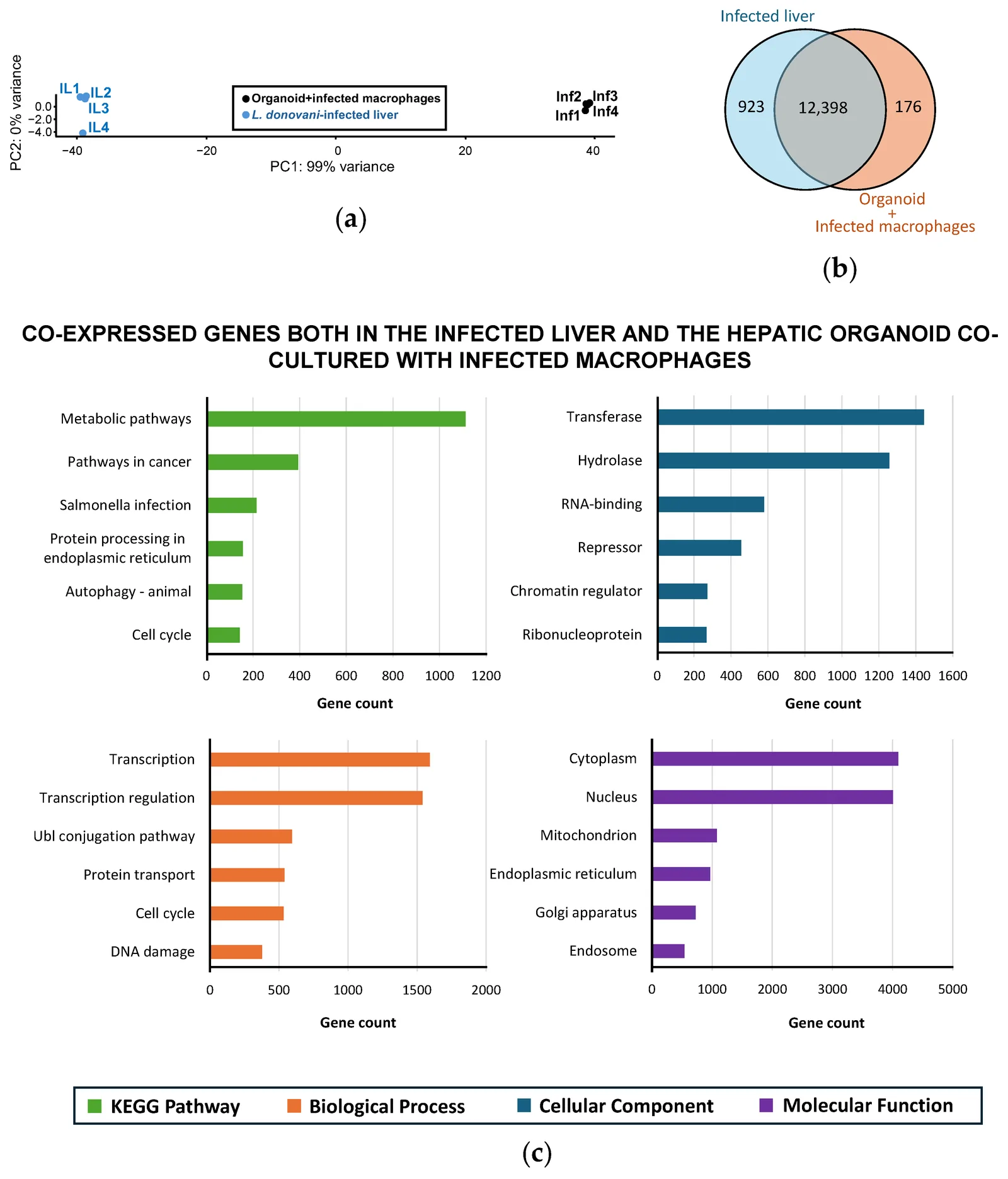
Comparative transcriptional analysis of *L. donovani*-infected liver and hepatic organoids co-cultured with *L. donovani*-infected macrophages: (**a**) Principal component analysis performed with samples of mouse hepatic organoids co-cultured with macrophages infected with *L. donovani*-mCh (Inf1-Inf4, black dots) and *L. donovani*-infected mouse liver (IL1-IL4, blue dots). (**b**) Venn diagram showing the transcripts detected in infected mouse liver tissue (blue circle) and in mouse hepatic organoids co-cultured with infected macrophages (orange circle). The number of genes co-expressed in both conditions is included in the intersection of the two circles. (**c**) Functional enrichment analysis of those transcripts from the 12,398 genes co-expressed in the infected liver and in the mouse hepatic organoid co-cultured with infected macrophages. The six most represented pathways in each category (KEGG pathway, biological process, cellular compartment, and molecular function) are represented as colored bars (only two chart records for the KEGG pathway, one chart record for the biological process and cellular component categories, and three chart records for the molecular function category were provided by the DAVID free software (v2024q4) during the analysis of those transcripts specific to the hepatic organoid co-cultured with infected macrophages).

To assess the ability of hepatic organoids co-cultured with infected macrophages to reproduce key aspects of *Leishmania* liver infection, a comparative transcriptomic analysis between the expression patterns observed in the experimentally infected in vivo liver tissue and those obtained from the in vitro model was performed. RNA-seq analysis was carried out, and principal component analysis revealed well-defined clusters separating both experimental models. This clear separation is primarily driven by Principal Component 1, which accounts for 99% of the total variance, highlighting the fundamental baseline differences between a complex in vivo microenvironment and the in vitro system (Figure 8a). The analysis showed that among the 13,497 detected transcripts, 923 (6.8%) were specific to the infected liver and 176 (1.3%) were unique to the hepatic organoid co-cultured with infected macrophages, while the vast majority (12,398 transcripts, 91.9%) were co-expressed in both conditions (Figure 8b).

Functional enrichment analysis was performed with the 12,398 genes co-expressed in the infected liver and in the 3D model, and the top six most significant metabolic pathways, based on the KEGG results, as well as biological, cellular, and molecular categories were identified (Figure 8c). Analysis revealed that metabolic pathways, pathways in cancer, and processes related to transcription and transcriptional regulation were overrepresented, the most active cellular sites are cytoplasm and nucleus, and transferases and hydrolases are highly abundant.

**Figure 9:**
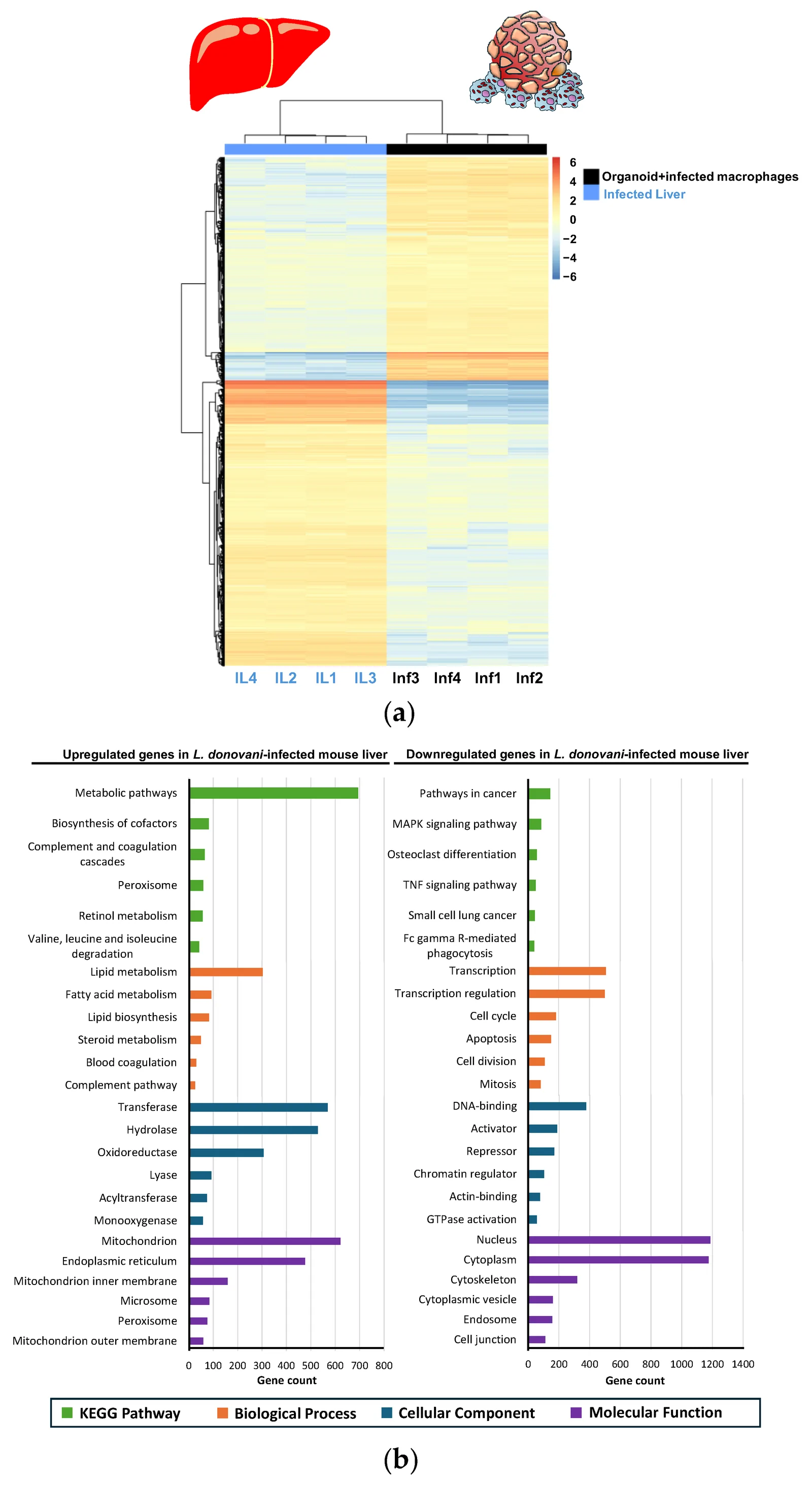
Differential expression between *L. donovani*-infected mouse livers and mouse hepatic organoids co-cultured with macrophages infected with *L. donovani*-mCh: (**a**) Heatmap showing the genes with the highest differential expression between mouse hepatic organoids co-cultured with macrophages infected with *L. donovani*-mCh (Inf1–Inf4) and mouse livers infected with *L. donovani*-PpyRE9h (IL1–IL4). The applied filters are: Log2FC < −1 or >1, and q-value < 0.1. Color scale indicates gene expression levels from low (blue) to high (red). Dendrograms reflect hierarchical clustering based on sample similarity. (**b**) Functional enrichment analysis of the differentially expressed genes in *L. donovani*-infected mouse livers in comparison with hepatic organoids co-cultured with *L. donovani*-infected macrophages. The left panel shows the analysis of the 4667 upregulated genes in the infected organ, while the right panel shows the analysis of the 3652 downregulated genes in the infected organ. The six most represented pathways in each category (KEGG pathway, biological process, cellular compartment, and molecular function) are represented as colored bars. The analysis was carried out with the DAVID free software (v2024q4).

Differential expression analysis revealed that, from the total of 13,497 detected transcripts in both conditions, 4677 genes were upregulated and 3652 downregulated in the infected liver compared to the co-culture of hepatic organoids with macrophages infected with *L. donovani*-mCh. Overexpressed and repressed genes can be visualized according to significant fold changes and statistical evidence in the heatmap (Figure 9a).

Functional enrichment analysis of the differentially expressed genes revealed the top six most significant metabolic pathways, based on the KEGG results, as well as biological, cellular, and molecular categories (Figure 9b). KEGG pathway analysis showed a strong enrichment of metabolic processes in infected liver tissue, with metabolic pathways representing the most prominent category. Consistently, UniProt biological process keywords highlighted enrichment in lipid and fatty acids metabolism. At the molecular function level, upregulated genes were predominantly associated with enzymatic activities characteristic of hepatocytes, including transferase, hydrolase, oxidoreductase, and monooxygenase functions. Cellular component analysis showed that upregulated genes were mainly localized to mitochondria, endoplasmic reticulum, microsomes, and peroxisomes, which are key organelles involved in energy metabolism, fatty acid β-oxidation, and xenobiotic metabolism. In contrast, downregulated genes in infected liver were enriched in KEGG pathways related to immune signaling and cellular communication. Biological process enrichment revealed a marked reduction in categories related to transcription and transcriptional regulation, proliferation and apoptosis. Accordingly, molecular function analysis showed enrichment for DNA-binding, chromatin regulator, activator, and repressor terms among downregulated genes. Finally, cellular component terms associated with downregulated genes were predominantly related to the nucleus and cytoplasm, cytoskeleton, cytoplasmic vesicles, and endosomes, compartments involved in intracellular trafficking, structural organization, and signal transduction (Figure 9b).

The six most upregulated and downregulated genes in the infected liver in comparison with the 3D in vitro model including infected macrophages are included in Table 7 and Table 8, respectively. The analysis revealed that *Leishmania*-infected liver tissue exhibits higher expression of genes related to metabolism, lipid transport, acute-phase response, and oxidative stress, including *Aldh2*, *Apoe*, *C3*, *Ndrg2*, *Orm1*, and *Selenop*, compared to hepatic organoids co-cultured with infected macrophages (Table 7). Conversely, genes such as *Ccl5*, *Ctss*, *Fth1*, *Gpnmb*, *Klf6*, *and Nfkbia*, which are involved in immune signaling, lysosomal function, and transcriptional regulation (Table 8), were downregulated in the liver relative to organoids.

Together, these findings highlight that the organoid co-culture model captures key aspects of hepatic infection, but it does not fully reproduce the complex in vivo microenvironment and immune responses observed during *Leishmania* infection.

**Figure 10:**
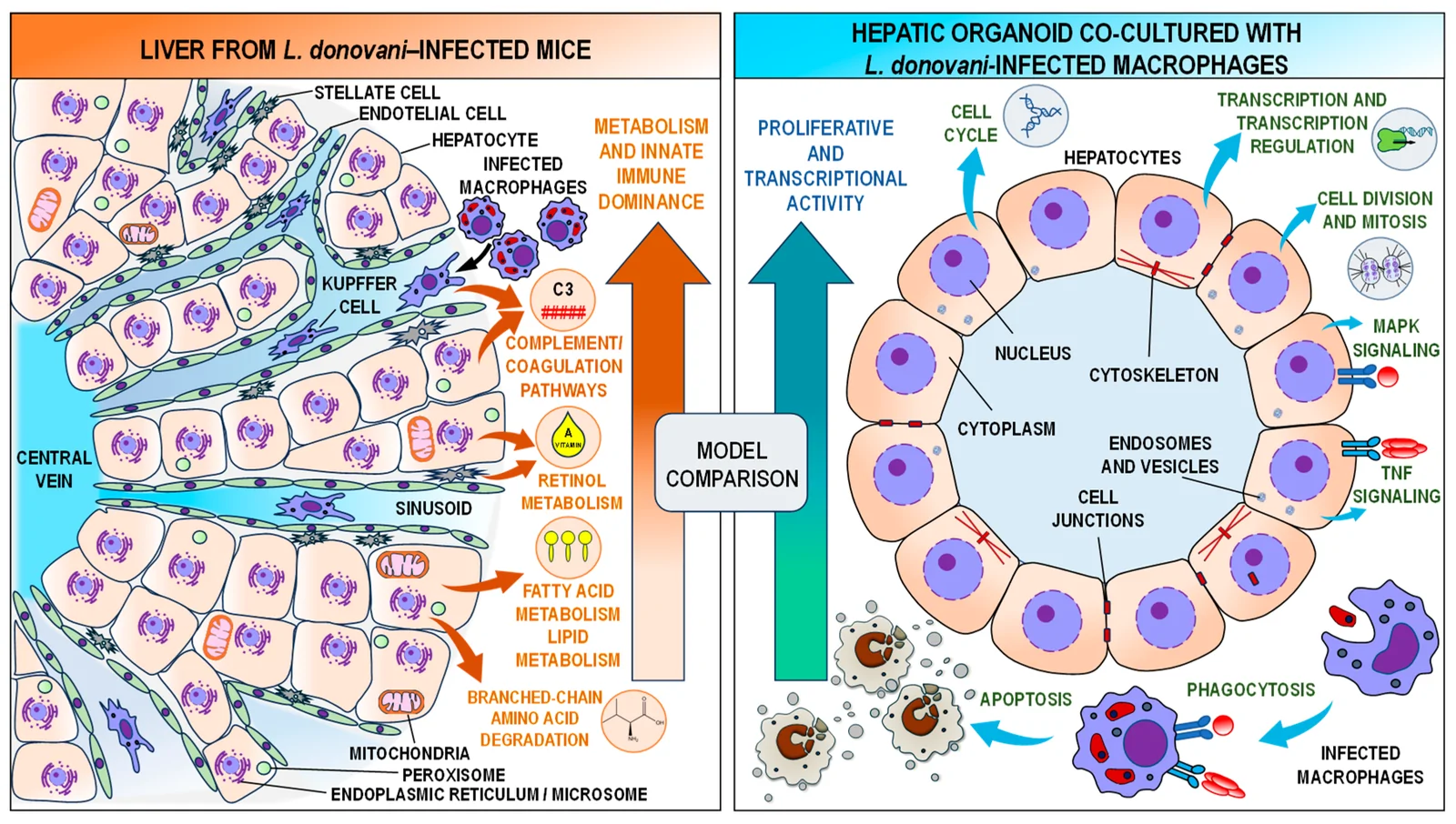
Comparative overview of in vivo liver and in vitro hepatic organoid co-culture responses to *L. donovani* infection. The main cellular components enriched in each model are also indicated.

In summary, both in vitro and in vivo systems share key features, including activation of inflammatory and chemokine signaling pathways and modulation of immune responses. However, important differences are observed: the in vivo liver exhibits broader immune activation, multicellular interactions, and pronounced metabolic reprogramming, particularly in lipid metabolism and acute-phase responses; whereas the organoid model shows enhanced transcriptional and proliferative activity and lacks systemic and vascular components (Figure 10). This comparison highlights the ability of the organoid system to capture core immune mechanisms while underscoring its limitations in modeling the full complexity of the hepatic microenvironment during infection.

## 3. Discussion

In this study, we combined in vitro organoid models with in vivo liver transcriptomic data to obtain an integrated view of the hepatic response to *L. donovani* infection. Rather than providing a population-level characterization of hepatic infection, the in vivo transcriptomic dataset served as a reference framework against which the immunocompetent organoid model could be evaluated. By integrating cytokine profiling and RNA sequencing analyses, we found that macrophages play a central role in shaping hepatic immune responses and that *L. donovani* infection induces a coordinated but functionally imbalanced immune response. Importantly, comparative analyses indicate that macrophage-containing hepatic organoids capture essential immunological and transcriptional features observed in infected liver tissue, supporting their value as a physiologically relevant experimental platform.

Organoid-based systems have emerged as powerful 3D platforms to model host–pathogen interactions under physiologically relevant conditions. By preserving key aspects of tissue architecture, cellular differentiation, and spatial organization, organoids enable the study of infection dynamics in a context that more closely resembles in vivo biology than conventional monolayer 2D cultures [30,31]. In recent years, their application has expanded to a wide range of infectious diseases, providing insights into pathogen tropism, immune responses, and tissue-specific mechanisms of pathogenesis [20,32,33]. Importantly, the incorporation of immune components, such as macrophages, represents a critical step toward enhancing the physiological relevance of these models and bridging the gap between in vitro systems and in vivo complexity. Our group previously developed mouse hepatic organoids and established a model of host–pathogen interaction for *Leishmania* [27], although the lack of cells from the immune system limited the ability of the 3D model to fully recapitulate the complexity of the hepatic immune microenvironment and host defense responses. To address these limitations, bone marrow-derived macrophages were co-cultured with hepatic organoids to develop an immunocompetent in vitro system, where some macrophages were binucleated, in agreement with previous reports showing that GM-CSF can promote macrophage fusion and multinucleated giant cell formation [34]. Macrophage multinucleation is a well-described process associated with cell–cell fusion [35]. In our model, macrophages displayed an elongated morphology typical of adherent cells under basal conditions, suggesting that multinucleation does not necessarily reflect a fully activated inflammatory state but may instead arise from differentiation and culture-dependent processes. Addition of macrophages to the hepatic organoids endowed the co-culture with functional characteristics consistent with a Kupffer cell-like phenotype. Although canonical Kupffer cell markers such as CLEC4F, TIM4 and VSIG4 were not evaluated, the cytokine profile, immunoregulatory features and epithelial–immune crosstalk observed in the co-culture support the acquisition of several functional properties associated with liver-resident macrophages rather than definitive Kupffer cell identity. These liver-resident macrophages play a critical role in maintaining immune surveillance while preserving tissue homeostasis [3,36]. Macrophage-containing organoids exhibited increased secretion of pro-inflammatory cytokines such as IL-1β, IL-6, and TNF, consistent with the primed basal inflammatory state described for Kupffer cells [4,37]. Importantly, this inflammatory profile was accompanied by a concomitant rise in IL-10 and IL-27, indicating that inflammatory activation is counterbalanced by regulatory mechanisms. Such dual functionality is a hallmark of Kupffer cells, which limit excessive inflammation to preserve hepatic homeostasis [38]. Additional features further supported the acquisition of a Kupffer-like phenotype by the incorporated macrophages. The chemokine profile suggested a stable resident macrophage population, as indicated by reduced CCL2 and CSF1 levels, which are typically associated with monocyte recruitment and differentiation [4]. In contrast, increased expression of CCL17 and CCL22 pointed toward roles in immune modulation and tissue repair, which aligns with the association of these chemokines with alternatively activated macrophages and with the established role of Kupffer cells in shaping the hepatic immune niche and maintaining tissue homeostasis [39,40,41]. The detection of IFN-γ and IFN-γ-inducible chemokines under basal conditions further supports the presence of functional innate immune signaling pathways in this model. Additional features reinforce the physiological relevance of the system. The chemokine profile is consistent with a stable population of differentiated macrophages, while the production of HGF and IL-22 suggests activation of tissue repair programs accompanying the inflammatory response, consistent with the coordinated regenerative responses described in the liver during chronic inflammation [36,42]. Moreover, the upregulation of immune checkpoint molecules such as PD-L1 and PD-L2 highlights the capacity of the model to capture immunoregulatory mechanisms characteristic of the hepatic microenvironment. This observation is consistent with previous reports describing the role of immune checkpoint-mediated regulation in shaping the tolerogenic immune landscape of the liver [43,44,45,46,47]. Collectively, these findings support the organoid–macrophage co-culture as a robust and physiologically relevant in vitro system to study liver-specific immune responses.

Upon infection, this model revealed a consistent pattern across cytokine and transcriptomic datasets characterized by activation of inflammatory pathways together with selective suppression of protective immune responses. The increased expression of *Il1a* and *Il1b* is consistent with inflammasome activation in infected macrophages. Prior studies have demonstrated that *Leishmania* species can activate NLRP3-dependent IL-1β production, contributing to local inflammation while not necessarily promoting parasite clearance [48,49]. In tissue contexts, IL-1 signaling may enhance leukocyte recruitment and vascular permeability, potentially expanding the pool of permissive host cells [50]. The concomitant elevation of CCL2, CCL4, and CCL5 supports this model, as these chemokines recruit monocytes and lymphocytes to infected sites [51]. Upon differentiation into macrophages, recruited monocytes serve as new cellular niches for *Leishmania*, thereby paradoxically facilitating parasite propagation [52].

Despite this inflammatory activation, key pathways required for effective parasite clearance were markedly suppressed. In particular, the IL-12–IFN-γ axis was consistently downregulated at both transcriptional and protein levels, including reduced production of IL-12, IL-27, IFN-γ, and TNF. This pathway is essential for macrophage activation and intracellular parasite killing, and its inhibition is a well-established immune evasion mechanism in leishmaniasis [53,54]. The concomitant downregulation of CXCL9 further suggests disruption of Th1 cell recruitment and feedback loops required to sustain protective cellular immunity [55]. Overall, cytokine production in the infected organoid model showed good agreement with the transcriptomic profile of cytokine-encoding genes. Nevertheless, some mediators, such as TNF and CXCL2, exhibited differences between mRNA abundance and protein secretion. Such discrepancies are not unexpected, as cytokine production is regulated at multiple levels beyond transcription, including mRNA stability, translational efficiency, secretion kinetics and protein turnover. In agreement with this interpretation, the transcriptomic analysis identified the induction of several well-established post-transcriptional and negative regulators of inflammatory responses, including members of the ZFP36 family, DUSP phosphatases and TNFAIP3, which are known to limit cytokine translation and release during sustained innate immune activation [56,57,58]. In addition, IFN-stimulated genes and *Stat1* and *Stat2*, which are key mediators of type I and type III IFN signaling [59], were also downregulated, indicating impaired IFN-mediated responses. Together, both datasets reveal a tightly regulated inflammatory state in which early innate activation coexists with selective suppression of effector cytokine release, favoring tissue protection and parasite persistence. This immunomodulation is selective: while inflammatory signaling is maintained or enhanced, antiviral sensors, inflammasome components, and genes involved in cell death pathways are repressed, and negative regulators of inflammation, including *Nfkbia*, *Tnfaip3*, and *Socs* family members, are upregulated. These findings suggest that *Leishmania* orchestrates a controlled inflammatory environment that balances host inflammation with suppression of critical antimicrobial pathways, contributing to parasite persistence and disease progression.

Comparative analyses highlight the central role of macrophages in shaping the hepatic response to infection. In the absence of macrophages, hepatic organoids primarily exhibited epithelial stress responses and impaired immune activation, including reduced antigen presentation and lysosomal function. In contrast, the presence of macrophages was associated with enrichment of pathways related to key immune pathways, including phagosome and lysosome activity, chemokine signaling, and canonical pathways associated with *Leishmania* infection. These findings indicate that macrophages are essential for an effective immune response, but also represent the primary cellular niche exploited by the parasite [60].

The in vivo transcriptomic analysis further revealed a more complex and integrated host response. Infected liver tissue showed strong activation of immune pathways, including antigen presentation, IFN signaling, and chemokine production, consistent with recruitment and activation of multiple immune cell populations. At the same time, a marked repression of metabolic pathways, particularly those related to lipid metabolism and xenobiotic processing, was observed. This metabolic shift likely reflects a redistribution of hepatic resources toward immune defense, as previously described in systemic infections [61].

Direct comparison between in vivo and in vitro systems demonstrated both convergence and divergence, highlighting both the power and the inherent limitations of 3D modeling. The profound separation observed in the principal component analysis, where PC1 accounted for 99% of the variance, underscores the inherent biological differences between a whole organism and an in vitro co-culture system. Despite these expected differences, the substantial overlap in transcriptional programs supports the biological relevance of the model. The pathways uniquely represented in vivo likely reflect systemic metabolic regulation and multicellular interactions that remain beyond the scope of current organoid systems. While the organoid model successfully captures key features of inflammatory and chemokine signaling, it did not fully reproduce the metabolic reprogramming and systemic responses observed in infected liver tissue. Genes related to lipid metabolism, acute-phase response, and oxidative stress were more prominently expressed in vivo, reflecting the contribution of hepatocyte-specific functions and systemic regulatory signals. Conversely, organoids showed relatively higher activity in pathways related to transcription and proliferation, likely due to the absence of systemic constraints and the simplified microenvironment. This is consistent with previous reports showing that current liver organoid systems, while capable of modeling key features of organotypic structure and some metabolic activities, do not fully recapitulate the complete functional complexity and systemic responses of in vivo liver tissue, including mature metabolic zonation and full hepatocyte phenotypes [62,63].

These findings highlight the strengths and limitations of the organoid model. The hepatic organoid–macrophage co-culture system captures essential aspects of host–pathogen interactions, including immune activation and parasite-driven immune modulation, but does not fully reproduce the complexity of the in vivo hepatic environment. In particular, the absence of vascularization, and systemic metabolic cues limits its ability to model whole-organ responses. Although the organoids capture key epithelial components of the liver, including hepatocyte- and cholangiocyte-lineage cells [27], they do not incorporate other non-parenchymal cell populations such as liver sinusoidal endothelial cells, hepatic stellate cells, or additional resident immune cells. These cell types contribute to immune regulation, tissue remodeling and intercellular signaling in vivo and their absence should be considered when interpreting the immune responses observed in the present model. Furthermore, we acknowledge important limitations regarding the in vivo transcriptomic profiling. The RNA-seq dataset was generated from liver sections obtained from a single infected and a single control mouse. Although multiple spatial sections were analyzed to capture intra-organ heterogeneity associated with hepatic infection, these samples do not represent independent biological replicates and therefore do not permit assessment of inter-individual variability. Consequently, the in vivo transcriptomic dataset should be regarded as a reference benchmark for comparison with the immunocompetent organoid model rather than as a population-level analysis. Future studies including larger animal cohorts will be required to validate the generalizability of the transcriptomic signatures identified here and to further characterize systemic metabolic adaptations during infection.

## 4. Materials and Methods

### 4.1. Culture of Mouse Hepatic Organoids

Mouse hepatic organoids were obtained from hepatic progenitor cells of BALB/c mice as previously described [27]. Briefly, livers were collected post-mortem, minced, and digested using a tissue dissociation protocol. The resulting cell suspension was filtered, pelleted, and embedded in Matrigel^®^ matrix (Corning^®^, Discovery Labware Inc., Bedford, MA, USA) in 24-well plates. Domes were overlaid with HepatiCult™ Mouse Organoid Growth Medium (HepatiCult™ mouse OGM, StemCell Technologies™, Vancouver, BC, Canada) and cultured at 37 °C with 5% CO_2_, with medium changes every 2–3 days. Organoids were passaged weekly by disrupting the matrix in ice-cold Advanced DMEM/F-12 (Gibco, Life Technologies Limited, Paisley, UK) by gentle pipetting and re-plating the pelleted cells following the same procedure.

### 4.2. Isolation and Culture of Bone Marrow-Derived Macrophages

Bone marrow was obtained from a healthy adult BALB/c mouse (8–10 weeks) following euthanasia performed according to institutional guidelines. Limbs were dissected, surface-decontaminated, and transferred to a sterile Petri dish. Soft tissues were removed to expose the femurs and tibias, which were then isolated. The epiphyses of each bone were excised to expose the medullary cavity, where red marrow was visibly present. Each bone was placed in a perforated 0.6 mL microfuge tube nested within a 1.5 mL microfuge tube containing 100 μL of supplemented RPMI 20/30 medium, consisting of RPMI medium (Gibco, Fisher Scientific International Inc., Pittsburgh, PA, USA) supplemented with 20% Fetal Bovine Serum (FBS), 30% Macrophage and Granulocyte Colony Stimulating Factor (GM-CSF) obtained from the supernatants of L929 cells, and 100 U/mL penicillin and 100 µg/mL streptomycin (antibiotic cocktail). Bone-marrow was released into the medium through controlled centrifugation at 600 g during 30 s. Cell suspensions from all four bones (two femurs and two tibias) were pooled and counted. A total of 6 × 10^6^ bone marrow cells were seeded onto Petri dishes in 10 mL of RPMI 20/30 medium and cultured at 37 °C and 5% CO_2_ to promote differentiation. On day 4, an additional 10 mL of RPMI 20/30 medium was added to the culture, and on day 7, bone marrow-derived macrophages were harvested and counted.

### 4.3. Infection of BALB/c Mice with Genetically Engineered L. donovani Strains and In Vivo/Ex Vivo Imaging

Genetically engineered strains of *L. donovani* LV9 were previously generated in our laboratory and were used for different purposes in this work. *L. donovani*-iRFP expresses the gene encoding the near-infrared fluorescent protein (iRFP) of *Rhodopseudomonas palustris* [64,65] and was used in the experiment comprising the co-culture of hepatic organoids with axenic amastigotes. Viable *L. donovani*-mCh amastigotes, which express the gene encoding the red fluorescent protein mCherry [66], were used to visualize intramacrophage parasites in the co-culture of hepatic organoids with infected macrophages. Finally, *L. donovani*-PpyrE9h, which expresses the firefly red-shifted luciferase PpyRE9h gene [67], was used to evaluate light emission by infected organs during in vivo experiments. These strains do not differ from one another in phenotype or behavior, except for the production of the different reporter proteins. Promastigotes of these strains were grown at 26 °C in Schneider’s insect medium (Sigma-Aldrich, Merck, Darmstadt, Germany) supplemented with 20% (*v*/*v*) FBS and antibiotic cocktail, until the mice were infected.

Female BALB/c (8 weeks old) mice were infected intraperitoneally with 1.5 × 10^9^ metacyclic promastigotes per mouse. Infections were maintained for 8 weeks.

For those mice infected with *L. donovani*-PpyrE9h, parasite burden was quantified by bioluminescence imaging. To record images, BALB/c mice were anesthetized with 1.5% (*v*/*v*) isoflurane (Perkin Elmer, Shelton, CT, USA) in O_2_, and after intraperitoneal injection of 50 µL of 30 mg/mL D-luciferin (PerkinElmer, Shelton, CT, USA), animals were allowed to distribute the substrate for 10 min and then placed in an IVIS Spectrum (PerkinElmer, Shelton, CT, USA) prewarmed to 36 °C. Bioluminescence images were acquired after 2 min. After image acquisition, the mice were humanely euthanized, and the liver and spleen were dissected and incubated for 10 min in 10 mL of PBS (Phosphate-buffered saline) containing 0.3 mg/mL D-luciferin. Living Image software version 4.5.4 (PerkinElmer) was used to quantify bioluminescence, expressed as total flux (photons/s), in Regions of Interest (RoIs) drawn around the entire animal [68].

### 4.4. Isolation and Culture of L. donovani-iRFP and L. donovani-mCh Amastigotes from Infected Mice

Eight weeks post-infection, mice infected with *L. donovani*-iRFP and *L. donovani*-mCh were euthanized. Femurs and tibias were aseptically collected, and bone marrow was obtained as indicated above. Bone-marrow derived cells were resuspended in 20 mL of amastigote medium (15 mM KCl, 136 mM KH_2_PO_4_, 10 mM K_2_HPO_4_·3H_2_O, 0.5 mM MgSO_4_·7H_2_O, 24 mM NaHCO_3_, 22 mM glucose, 1 mM L-glutamine, 1× RPMI 1640 vitamins, 10 μM folic acid, 100 μM adenosine, 1× RPMI 1640 amino acids, 5 μg/mL hemin, antibiotic cocktail, and 25 mM MES in Milli-Q water, adjusted to pH 5.66 and supplemented with 10% FBS). Bone marrow cells were incubated at 37 °C with 5% CO_2_ to release the axenic amastigotes from macrophages, which were subsequently maintained under the same conditions.

### 4.5. In Vitro Infection of Bone Marrow-Derived Macrophages with L. donovani-mCh

Since maintaining cultures of infected macrophages isolated from mice is challenging, infected macrophages were generated by in vitro infection of primary macrophages with amastigotes derived from infected mice. With this purpose, a total of 0.5 × 10^6^ bone marrow-derived macrophages obtained as indicated above, were plated into 24-well plates and allowed to adhere for 2 h at 36 °C and 5% CO_2_ in RPMI 10/15, which included RPMI medium supplemented with 10% FBS, 15% GM-CSF from L929 cells, and antibiotic cocktail. Macrophages were then exposed for 24 h to 2 × 10^6^ *L. donovani*-mCh amastigotes obtained from bone marrow-derived cells extracted from experimental infections as indicated above.

### 4.6. Liver Sampling from Healthy and L. donovani-PpyrE9h-Infected BALB/c Mice and Histological Assessment

Infected BALB/c mice (16 weeks of age, 8 weeks post-infection with *L. donovani*-PpyrE9h) and age-matched uninfected female BALB/c controls were euthanized, and their livers were aseptically removed. The presence of living *L. donovani*-PpyrE9h in the explants was quantified by bioluminescence imaging using an IVIS Spectrum as indicated above. For the in vivo transcriptomic analysis, to capture spatial intra-organ responses, a representative liver from each group (one infected and one healthy) was sectioned into four distinct pieces (~1 cm^2^ each). These spatial replicates were immediately stored in RNAlater^®^ (Fisher Scientific, Waltham, MA, USA) at −80 °C for subsequent RNA isolation and RNA-seq analysis. Additionally, the distal portion of the left lateral liver lobe from the infected mouse was collected for histological assessment. This sample was fixed in 10% neutral buffered formalin, embedded in paraffin wax, sectioned, and stained with hematoxylin and eosin. Micrographs were acquired using a Leica^®^ DM2000 microscope coupled with an ICC50W digital camera (Leica Microsystems, Wetzlar, Germany).

### 4.7. Co-Culture with Hepatic Organoids

Co-culture of hepatic organoids with axenic *L. donovani*-iRFP amastigotes was performed in 24-well plates as previously described [27]. Briefly, after 7 days of organoid culture, the Matrigel^®^ matrix (Corning®, Discovery Labware Inc., Bedford, MA, USA) was gently dissociated using Advanced DMEM/F-12, and cells were collected by centrifugation. The cell pellet was resuspended in Matrigel^®^ containing previously washed axenic *L. donovani*-iRFP amastigotes (2 × 10^6^ per well), obtained from bone marrow-derived cells as indicated above. Co-cultures were incubated for 24 h at 37 °C with 5% CO_2_.

To establish hepatic organoid-macrophage co-cultures, a total of 0.5 × 10^6^ bone marrow-derived macrophages (either uninfected or infected with *L. donovani*-mCh) were plated onto 24-well plates and allowed to adhere in RPMI 10/15 (see above) at 36 °C and 5% CO_2_. After 2 h, the medium was removed, and hepatic organoids were added as follows. Organoids were cultured according to the protocol previously described [27], dissociated with 800 μL of pre-warmed Advanced DMEM/F-12 and reconstituted as three-dimensional domes using 35 μL of Matrigel^®^ matrix per well directly on top of the infected macrophage monolayer, establishing the co-culture system. Co-cultures were maintained for an additional 24 h.

Supernatants were collected for cytokine quantification and stored at −20 °C, and cell pellets were harvested and centrifuged at 290× *g* for 5 min at 4 °C, resuspended in RNAlater^®^, and stored at −80 °C for subsequent RNA isolation and RNA-seq analysis.

### 4.8. RNA-Seq and Transcriptomics Analysis

Frozen material, either the co-culture of organoids or liver tissue, was sent to Seqplexing Genetest (Valencia, Spain) for RNA isolation and transcriptomics analysis. The experimental design included four biological replicates for each condition. All RNA samples passed sample quality control. RNA-seq libraries were subsequently generated and sequenced on an Illumina NovaSeq X system (Illumina, Inc., San Diego, CA, USA) using a paired-end 2 × 150 bp configuration. Bioinformatic processing of the raw reads was carried out to ensure data quality. Quality control checks confirmed appropriate fragment length distribution, and sequencing quality metrics (including read counts, GC content, and Q20/Q30 scores) were obtained using FastQC v0.11.9. Reads were aligned to the mouse reference genome GRCm39 with STAR v2.7.10, allowing for splice-aware mapping, and duplicates were removed using UMI-tools. Gene-level read counts were obtained with HTSeq-count v2.0.2, and mapping efficiency exceeded 90% in most samples. Expression values were normalized using DESeq2 v1.38.0, and differential expression analysis was performed with multiple testing correction via the Benjamini–Hochberg FDR method. Data visualization and plotting were conducted in R (v4.4.2) using ggplot (v3.5.1) and pheatmap (v1.0.12) to generate heatmaps, volcano plots, PCA graphs, and dispersion plots.

Genes were considered significantly differentially expressed when Log2FC was greater than 1 (upregulated) or less than −1 (downregulated) with an adjusted q-value below 0.1. Gene identifiers were converted to ENTREZ Gene IDs, with a conversion success rate above 99%.

Functional enrichment analyses were performed using the DAVID Functional Annotation tool (NIH) [69,70] to explore KEGG pathways and gene ontology categories (biological process, cellular component, and molecular function).

### 4.9. Cytokine Analysis

Supernatants from the hepatic organoid cultures and co-cultures were analyzed using the Olink^®^ Target 48 Mouse Cytokine Panel (Olink Proteomics, Uppsala, Sweden). The measurements were conducted by Cobiomic Bioscience (Córdoba, Spain), and each experimental condition was analyzed in three technical replicates. The panel provided results for 42 cytokines.

### 4.10. Immunofluorescence Staining and Microscopy

For imaging, the different co-cultures were established in 8-well Lab-Tek chamber slides (Thermo Fisher Scientific, Rochester, NY, USA) at the same relative seeding densities and infection ratios used for 24-well plates.

To avoid iRFP fluorescence loss after fixation, Matrigel^®^ domes containing hepatic organoids co-cultured with axenic *L. donovani*-iRFP amastigotes were mounted fresh using Mount Quick Aqueous (05-1740, Bio Optica, Milano, Italy) and imaged within 30 min (#1 coverslips were used as spacers to prevent compression). Micrographs were acquired using an Eclipse Ni-E (Nikon, Tokyo, Japan) equipped with the Prime BSI Scientific CMOS monochrome scientific camera (Photometrics^®^ Prime BSI™, Scottsdale, AZ, USA) for the iRFP epifluorescence channel, and the DS-Ri2 color microscope camera (Nikon, Tokyo, Japan) for the brightfield channel. A Z-stack was acquired with a 0.5 µm step size, and automatic field-of-view alignment followed by a maximum intensity projection, was performed using Nikon NIS-Elements Advanced Research software (version 6.20.00).

The immunofluorescence staining of either infected or uninfected macrophages, and its co-culture with hepatic organoids, was adapted from previously described protocols [27,71,72]. Briefly, after 24 h of culture with or without *L. donovani*-mCh, bone-marrow derived macrophages were washed three times with PBS and fixed with methanol-free paraformaldehyde (Thermo Fisher Scientific, Rochester, NY, USA) at a concentration of 4% in PBS. After washing, permeabilization and blocking were performed with 0.2% Triton™ X-100 (Sigma-Aldrich, Merck, Darmstadt, Germany) and 3% bovine serum albumin (BSA) (Roche Diagnostics, Mannheim, Germany) in PBS for 1 h at 37 °C. Afterwards, an overnight incubation at 4 °C, with the rabbit anti-Iba1 (ionized calcium-binding molecule 1) (019–19741, Wako, Japan; 1:500) was performed. Then, after washing, samples were incubated with the secondary antibody goat Anti-Rabbit IgG H&L (Alexa Fluor 488) (ab150077, Abcam (Cambridge, UK; 1:500). PBS with 3% BSA was used for antibody dilutions. Finally, the Lab-Tek chamber was removed, and samples were mounted using Fluoroshield™ with DAPI (4′,6-diamidino-2-phenylindol) (Sigma-Aldrich, Merck, Darmstadt, Germany). Images were taken using the microscope Eclipse Ni-E equipped with the Prime BSI Scientific CMOS scientific camera.

For staining of macrophages and hepatic organoids inside the Matrigel^®^ matrix, after 24 h of co-culture, prewarmed 16% methanol-free paraformaldehyde was added directly to the culture medium to a final concentration of 4% to preserve the integrity of the domes, and fixation was performed at 37 °C for 1 h. All washes were performed with prewarmed PBS at 37 °C. After washing, samples were permeabilized with 0.2% Triton™ X-100 for 30 min at 37 °C, followed by additional washing steps, and blocking with 3% BSA in PBS for 2 h at 37 °C. Samples were then incubated overnight at 4 °C with rat anti-ZO-1 (R40.76, Santa Cruz Biotechnology, Dallas, TX, USA; 1:50) and rabbit anti-Iba1 (1:500). Subsequently, after washing, samples were incubated with goat anti-rabbit IgG (H&L, Alexa Fluor 488; 1:500) for 2 h at room temperature. After six 10 min washes at 37 °C, the Lab-Tek chamber was removed, and samples were mounted using Fluoroshield™ with DAPI. #1 coverslips were used as spacers to minimize compression. Additionally, confocal imaging was performed on a Zeiss LSM 800 (Observer Z1, Carl Zeiss, Oberkochen, Germany). Z-stack images were acquired at intervals of 0.25 µm. All images were subjected to background subtraction prior to further processing. Deconvolution was performed using a regularized inverse filter for the organoids co-cultured with non-infected macrophages and a nearest neighbor algorithm for the co-culture with *L. donovani*-mCh-infected macrophages. Three-dimensional reconstruction was carried out using ZEN 2.6 software (Blue Edition, v2.6.76.00000; Carl Zeiss, Oberkochen, Germany; file v2.6.18299.3).

### 4.11. Ethics Declarations

All animal experiments complied with Spanish and European Union regulations (RD 53/2013 and 2010/63/EU) and were approved by the institutional Ethics Committee (Project license OEBA-ULE-010-2023).

### 4.12. Statistics

Statistical analysis of cytokine data was performed using SigmaPlot^®^ version 10.0 (Grafiti LLC, Austin, TX, USA). Comparisons between groups were conducted using an unpaired, two-tailed Student’s *t*-test. Statistical significance was defined as *p* ≤ 0.05. Statistical analysis for transcriptomics analysis is included within Section 4.8. Statistical parameters and tests are reported in the Figures and corresponding Figure Legends.

## 5. Conclusions

Overall, our findings support a model in which *L. donovani* infection is associated with a conserved pattern of hepatic immune dysregulation, characterized by simultaneous inflammatory activation and suppression of antimicrobial immune pathways. The hepatic organoid–macrophage system provides a tractable and scalable platform for investigating key aspects of these processes and may support the development of host-directed therapeutic strategies for VL.

## Figures and Tables

**Table 1:** Top six most overexpressed genes in mouse hepatic organoids in co-culture with *L. donovani*-mCh infected macrophages, ranked by *p*-value.

Gene	Description ^1^	Log2FC ^2^	*p*-Value
*Dusp1*	Dual specificity phosphatase 1. Involved in negative regulation of MAPK cascade.	2.9	3.9 × 10^−77^
*Hsph1*	Heat shock 105kDa/110kDa protein 1. Involved in chaperone cofactor-dependent protein refolding.	3.3	2.3 × 10^−65^
*Cxcl2*	C-X-C motif chemokine ligand 2. Involved in chemokine activity; response to bacterial molecules.	2.5	1.5 × 10^−57^
*Saa1*	Serum amyloid A 1. G protein-coupled receptor binding activity: Involved in cholesterol metabolism and response to bacteria.	4.6	6.8 × 10^−57^
*Il1b*	Interleukin 1 beta. Involved in thymocyte proliferation and inflammatory response.	2.8	2.1 × 10^−50^
*Hp*	Haptoglobin. Sequestration and clearance of extracorpuscular hemoglobin.	1.7	1.4 × 10^−46^

^1^ Information retrieved from the National Library of Medicine (https://www.ncbi.nlm.nih.gov/datasets/gene/ accessed on 10 January 2026). ^2^ Log2FC = Log_2_ fold change.

**Table 2:** Top six most downregulated genes in mouse hepatic organoids in co-culture with *L. donovani*-mCh infected macrophages, ranked by *p*-value.

Gene	Description ^1^	Log2FC ^2^	*p*-Value
*Ifit2*	Interferon-induced protein with tetratricopeptide repeats 2. Involved in response to antiviral innate immune and to interferon-alpha.	−5.0	7.5 × 10^−87^
*Rsad2*	Radical S-adenosyl methionine domain containing 2. Involved in T cell differentiation, immune response and negative regulation of viral genome replication.	−2.8	3.7 × 10^−71^
*Ifit3*	Interferon-induced protein with tetratricopeptide repeats 3. Involved in cellular response to interferon-beta and bacteria.	−4.5	2.0 × 10^−64^
*Ifi44*	Interferon-induced protein 44. Involved in cellular response to virus and bacteria.	−2.6	1.2 × 10^−53^
*Isg15*	ISG15 ubiquitin-like modifier. Involved in ISG15-protein conjugation; defense response to other organisms.	−2.3	2.8 × 10^−51^
*Oasl1*	2′-5′ oligoadenylate synthetase-like 1. Involved in interleukin-27-mediated signaling pathway.	−2.3	3.7 × 10^−44^

^1^ Information retrieved from the National Library of Medicine (https://www.ncbi.nlm.nih.gov/datasets/gene/ accessed on 10 January 2026). ^2^ Log2FC = Log_2_ fold change.

**Table 3:** Top six most upregulated genes in mouse hepatic organoids co-cultured with *L-donovani*-mCh-infected macrophages in comparison with mouse hepatic organoids co-cultured with *L. donovani*-iRFP, ranked by *p*-value.

Gene	Description ^1^	Log2FC ^2^	*p*-Value
*B2m*	Beta-2 microglobulin. Component of MHC class I complexes involved in antigen presentation.	5.0	0.0
*Fth1*	Ferritin heavy polypeptide 1. Involved in iron transport.	5.1	0.0
*H2-D1*	Histocompatibility 2, D region locus 1. MHC class I molecule involved in presentation of endogenous peptide antigens in a TAP-dependent manner.	4.0	1.0 × 10^−286^
*Ctsb*	Cathepsin B. Lysosomal cysteine protease involved in protein degradation and turnover.	4.7	6.9 × 10^−269^
*Ctsd*	Cathepsin D. Lysosomal aspartic protease involved in protein degradation and autophagosome assembly.	3.0	1.2 × 10^−249^
*Ctsl*	Cathepsin L. Lysosomal cysteine protease (peptidase C1 family) involved in protein degradation and antigen processing.	4.4	4.6 × 10^−226^

^1^ Information retrieved from the National Library of Medicine (https://www.ncbi.nlm.nih.gov/datasets/gene/ accessed on 10 January 2026). ^2^ Log2FC = Log_2_ fold change.

**Table 4:** Top six most downregulated genes in mouse hepatic organoids co-cultured with *L- donovani*-mCh-infected macrophages in comparison with mouse hepatic organoids co-cultured with *L. donovani*-iRFP, ranked by *p*-value.

Gene	Description ^1^	Log2FC ^2^	*p*-Value
*Ccnd2*	Cyclin D2. Cyclin-dependent kinase activator. Involved in cell cycle regulation and cellular response to X-ray.	3.3	6.3 × 10^−280^
*Timp3*	Tissue inhibitor of metalloproteinase 3. Inhibition of metalloendopeptidase activity; zinc ion binding activity.	−3.6	6.3 × 10^−173^
*Cxcl5*	C-X-C motif chemokine ligand 5. Neutrophil recruitment via CXCR2; roles in inflammatory and homeostatic processes.	−2.6	3.6 × 10^−139^
*Ankrd1*	Ankyrin repeat domain 1. Involved in cellular response to lipopolysaccharide and cellular response to xenobiotic stimulus.	−3.2	6.0 × 10^−109^
*Dkc1*	Dyskeratosis congenita 1, dyskerin. Involved in rRNA modification (pseudouridylation); telomerase RNA binding; telomere maintenance.	−2.7	1.4 × 10^−108^
*Ccnd1*	Cyclin D1. Involved in regulation of G1/S cell cycle transition; transcriptional regulation; negative regulation of epithelial cell differentiation.	−1.9	3.5 × 10^−106^

^1^ Information retrieved from the National Library of Medicine (https://www.ncbi.nlm.nih.gov/datasets/gene/ accessed on 10 January 2026). ^2^ Log2FC = Log_2_ fold change.

**Table 5:** Top six most overexpressed genes in mouse livers infected with *L. donovani*-PpyrE9h, ranked by *p*-value.

Gene	Description ^1^	Log2FC ^2^	*p*-Value
*Cd74*	CD74 antigen (invariant polypeptide of major histocompatibility complex, class II antigen-associated). MHC class II chaperone involved in antigen processing and presentation; regulation of cytokine signaling and T cell differentiation.	5.1	0.0
*Ly6a*	Lymphocyte antigen 6 family member A. Acetylcholine receptor binding/inhibition involved in response to bacteria.	6.0	5.2 × 10^−284^
*H2-Eb1*	Histocompatibility 2, class II antigen E beta. Involved in antigen presentation.	5.6	2.2 × 10^−262^
*Iigp1*	Interferon inducible GTPase 1. GTPase activity; involved in defense response to bacteria and protozoa; regulation of autophagy; interferon-beta response.	2.2	1.5 × 10^−232^
*H2-DMb1*	Histocompatibility 2, class II, locus Mb1. Involved in MHC class II peptide loading; antigen presentation; chaperone-mediated protein folding.	4.7	7.6 × 10^−228^
*H2-Ab1*	Histocompatibility 2, class II antigen A, beta 1. Involved in antigen presentation.	5.2	4.6 × 10^−222^

^1^ Information retrieved from the National Library of Medicine (https://www.ncbi.nlm.nih.gov/datasets/gene/ accessed on 10 January 2026). ^2^ Log2FC = Log_2_ fold change.

**Table 6:** Top six most downregulated genes in mouse livers infected with *L. donovani*-PpyrE9h, ranked by *p*-value.

Gene	Description ^1^	Log2FC ^2^	*p*-Value
*Pzp*	PZP alpha-2-macroglobulin like 2. Endopeptidase inhibitor involved in embryo implantation.	−4.2	4.4 × 10^−273^
*Cyp2c69*	Cytochrome P450, family 2, subfamily c, polypeptide 69. Involved in xenobiotic and organic acid metabolism; monooxygenase activity.	−3.6	9.1 × 10^−130^
*Hamp2*	Hepcidin antimicrobial peptide 2. Peptide hormone synthesized in the hepatocytes that functions in the regulation of systemic iron metabolism.	−1.7	6.6 × 10^−101^
*Cyp4a14*	Cytochrome P450, family 4, subfamily a, polypeptide 14. Involved in fatty acid metabolism; eicosanoid biosynthesis; oxidoreductase activity	−5.5	4.4 × 10^−97^
*Cyp2a22*	Cytochrome P450, family 2, subfamily a, polypeptide 22. Involved in xenobiotic metabolism; epoxygenase P450 pathway; monooxygenase activity.	−3.1	1.3 × 10^−93^
*Acot3*	Acyl-CoA thioesterase 3. Involved in fatty acyl-CoA hydrolysis; lipid and carboxylic acid metabolism.	−2.8	3.8 × 10^−88^

^1^ Information retrieved from the National Library of Medicine (https://www.ncbi.nlm.nih.gov/datasets/gene/ accessed on 10 January 2026). ^2^ Log2FC = Log_2_ fold change.

**Table 7:** Top six most overexpressed genes in mouse livers infected with *L. donovani*-PpyrE9h in comparison with hepatic organoids co-cultured with *L. donovani*-mCh-infected macrophages, ranked by *p*-value.

Gene	Description ^1^	Log2FC ^2^	*p*-Value
*Aldh2*	Aldehyde dehydrogenase 2, mitochondrial. Aldehyde metabolism; NAD(P)-dependent oxidoreductase activity; regulation of dopamine and serotonin biosynthesis.	2.7	0.0
*Apoe*	Apolipoprotein E. Involved in the transport of lipoproteins in the blood; lipid metabolism; receptor-mediated lipid uptake.	3.1	0.0
*C3*	Complement component 3. Central component of complement cascade; immune response activation and opsonization.	2.6	0.0
*Ndrg2*	N-myc downstream regulated gene 2. Involved in negative regulation of ERK signaling; cytokine production; inhibition of vascular smooth muscle proliferation.	5.5	0.0
*Orm1*	Orosomucoid 1. Member of the lipocalin family of proteins. Acute-phase response protein synthesized by hepatocytes in response to cytokines; modulation of inflammation and metabolism.	5.2	0.0
*Selenop*	Selenoprotein P. Predominantly expressed in the liver and secreted into the plasma. Involved in selenium transport; extracellular antioxidant function; redox homeostasis.	3.5	0.0

^1^ Information retrieved from the National Library of Medicine (https://www.ncbi.nlm.nih.gov/datasets/gene/ accessed on 10 January 2026). ^2^ Log2FC = Log_2_ fold change.

**Table 8:** Top six most downregulated genes in mouse livers infected with *L. donovani*-PpyrE9h in comparison with hepatic organoids co-cultured with *L. donovani*-mCh-infected macrophages, ranked by *p*-value.

Gene	Description ^1^	Log2FC ^2^	*p*-Value
*Ccl5*	C-C motif chemokine ligand 5. Involved in chemokine signaling via CCR receptors; monocyte chemotaxis; regulation of inflammatory response; negative regulation of macrophage apoptotic process; positive regulation of epithelial cell proliferation.	−5.5	0.0
*Ctss*	Cathepsin S. Member of the peptidase C1 (papain) family of cysteine proteases. Antigen-processing protease; secreted cysteine protease in antigen-presenting cells; involvement in inflammatory signaling.	−3.6	0.0
*Fth1*	Ferritin heavy polypeptide 1. Involved in iron storage, transport and homeostasis.	−3.5	0.0
*Gpnmb*	Glycoprotein (transmembrane) nmb. Involved in modulation of tumor necrosis factor production; positive regulation of ERK signaling and protein phosphorylation.	−6.2	0.0
*Klf6*	Kruppel-like transcription factor 6. Involved in transcriptional regulation; cytokine-mediated signaling; connective tissue remodeling.	−4.9	0.0
*Nfkbia*	Nuclear factor of kappa light polypeptide gene enhancer in B cells inhibitor, alpha. Involved in inhibition of NF-κB signaling; regulation of immune and inflammatory transcriptional responses.	−3.1	0.0

^1^ Information retrieved from the National Library of Medicine (https://www.ncbi.nlm.nih.gov/datasets/gene/ accessed on 10 January 2026). ^2^ Log2FC = Log_2_ fold change.

## Data Availability

Data supporting reported results can be found in the Zenodo repository at https://doi.org/10.5281/zenodo.19510633.

## Supplementary Materials

The following supporting information can be downloaded at: https://www.mdpi.com/article/10.3390/ijms27167178/s1.

## Abbreviations

The following abbreviations are used in this manuscript:

2D	Two-dimensional
3D	Three-dimensional
BSA	Bovine serum albumin
FBS	Fetal bovine serum
iNOS	inducible Nitric Oxide Synthase
iRFP	Infrared fluorescent protein
KEGG	Kyoto Encyclopedia of Genes and Genomes
mCh	mCherry
PBS	Phosphate-buffered saline
VL	Visceral Leishmaniasis
ZO-1	Zonula occludens-1
