# Supplementary material for: An Immunocompetent Hepatic Organoid Model Reveals Conserved Hepatic Immune Dysregulation During Leishmania donovani Infection
Source: Int J Mol Sci. 2026 Aug 11;27(16):7178. doi: 10.3390/ijms27167178 (PMC13513185; doi:10.3390/ijms27167178)
Supplement: Supplementary file 1 [file ijms-27-07178-s001.zip › Supplementary Table S1.pdf]

Supplementary Table S1. Genes encoding cytokines and other proteins involved in the inflammatory process and immune response upregulated in hepatic organoids after co-culture with macrophages infected with *L. donovani*-mCh.

Immunological Process	Genes	Main Function
Proinflammatory Cytokines	*Il1a, Il1b, Il1f9, Tnf, Tnfsf15, Osm, Mif*	Induction and amplification of innate and adaptive inflammatory response
Cytokine Receptors and Signaling	*Il22ra1, Il9r, Il17rd, Tnfrsf10b, Tnfrsf23, Irak2, Malt1, Lyn*	Cellular response and intracellular inflammatory signaling
Chemokines and Leukocyte Recruitment	*Cxcl1, Cxcl2, Ccl3, Ccl4, Ccl8, Ccl12, Ccl24, Cx3cr1*	Chemotaxis and migration of immune cells
Complement and Opsonization	*C1qa, C1qb, C1qc, C4b, Cfp, Cfh, Cd55*	Pathogen elimination and tissue damage control
Pathogen Recognition (PRRs)	*Cd14, Lbp, Pglyrp1, Marco, Gp2*	Recognition of microbial patterns and innate immune activation
Direct Antimicrobial Defense	*Lcn2, Duox2, Olfm4, Slpi, Plac8*	Microbial growth restriction and tissue protection
Intracellular Inflammatory Signaling	*Map3k8, Card10, Card14, Rasgrp4, Trpm2, Sirpb1b*	Activation of NF-κB/MAPK pathways and cell signaling
Negative Regulation and Resolution	*Nfkbia, Nfkbiz, Nfkbid, Tnfaip3, Tnip2, Zc3h12a, Socs2, Dusp1, Dusp2, Dusp4, Dusp8, Nr4a1, Nr4a3*	Inflammatory response shutdown and fine control
Transcription Factors and Cell Differentiation	*Runx1, Bach2, Bcl6, Gata3, Rora, Tox, Sox4, Prdm16, Egr2, Bhlhe40, Cebpd*	Regulation of immune cell differentiation and function
Macrophages and Monocytes	*Ly6c2, Arg1, Mcemp1, Tmem176a, Inava, Vnn1, Vnn3, Mefv*	Identity, activation, and polarization of myeloid cells
Neutrophils and Acute Inflammation	*Ly6g2, Retn*	Rapid innate defense and microbial killing
Apoptosis, Cellular Stress, and Post-transcriptional Control	*Bik, Birc2, Dedd2, Pmaip1, Perp, Trp53inp1, Gadd45a, Gadd45b, Gadd45g, Fam162a, Mir155hg, Zfp36, Zfp36l1, Zfp36l2*	Cell death, damage response, and gene expression regulation
