# Supplementary material for: An Immunocompetent Hepatic Organoid Model Reveals Conserved Hepatic Immune Dysregulation During Leishmania donovani Infection
Source: Int J Mol Sci. 2026 Aug 11;27(16):7178. doi: 10.3390/ijms27167178 (PMC13513185; doi:10.3390/ijms27167178)
Supplement: Supplementary file 1 [file ijms-27-07178-s001.zip › Supplementary Table S2.pdf]

**Supplementary Table S2.** Genes encoding cytokines and other proteins involved in the inflammatory process and immune response downregulated in hepatic organoids after co-culture with macrophages infected with *L. donovani*-mCh.

| **Immunological Process** | **Genes** | **Main Function** |
|---|---|---|
| Proinflammatory Cytokines | *Il12b, Il15ra, Il21r, Il1rn, Tnfaip8l2* | Induction and amplification of innate and adaptive inflammation |
| Cytokine Receptors and Signaling | *Ikbip, Irf2bp1, Irf5, Irf9, Stat1, Stat2, Tfec, Themis2, Tecpr2, Trafd1, Pik3cd, Plcb2* | Cellular response and inflammatory signaling transduction |
| Chemokines and Leukocyte Recruitment | *Ccl2, Ccl7, Ccl24, Ccl25, Cxcl9, Cxcl10, Cxcl11, Csf3* | Chemotaxis and immune cell migration |
| Pathogen Recognition (PRRs) | *Cd300lf, Dhx58, Ddx58, Ifih1, Mavs, Naip2, Scara3, Slamf9, Treml2, Ticam2, Timd4, Tlr4, Tlr7, Tlr8, Tlr13, Zbp1* | Detection of pathogen-associated molecular patterns and innate immune activation |
| Direct Antimicrobial Defense | *Rsad2, Ifit1, Ifit2, Ifit3, Ifit3b, Ifi35, Ifi44, Ifi44l, Ifi47, Ifi202b, Ifi204, Ifi207, Ifi208, Oasl1, Oasl2, Oas1a, Oas1b, Oas1c, Oas1g, Oas3, Gbp2, Gbp2b, Gbp3, Gbp4, Gbp5, Gbp7, Gbp9, Isg15* | Microbial growth restriction and tissue protection |
| Intracellular Inflammatory Signaling | *Casp1, Pycard, Dapk3, Def6, Ecsit, Rtp4, Rinl, Usp18* | Activation of inflammatory and apoptotic pathways |
| Negative Regulation and Resolution | *Parp11, Parp12, Parp14, Usp18* | Inflammatory response shutdown and control |
| Transcription Factors and Cell Differentiation | *Batf2, Arid5a, Sp110* | Regulation of immune cell fate and function |
| Macrophages and Monocytes | *Gbp2, Gbp2b, Gbp3, Gbp4, Gbp5, Gbp7, Gbp9, Mrc1, Fermt3, Dock8, Dock10, Dok3, Elmo1* | Myeloid cell polarization, activation, and function |
| Neutrophils and Acute Inflammation | *Def6, Serpinb9, Csf3* | Rapid innate immune response and microbial killing |
| Apoptosis, Cellular Stress, and Post-transcriptional Control | *Bid, Bik, Btk, Pml, Fam162a, Mlkl, Mul1, Pml, Pycard, Batf2* | Cell death, stress response, and gene regulation |
