# Supplementary material for: An Immunocompetent Hepatic Organoid Model Reveals Conserved Hepatic Immune Dysregulation During Leishmania donovani Infection
Source: Int J Mol Sci. 2026 Aug 11;27(16):7178. doi: 10.3390/ijms27167178 (PMC13513185; doi:10.3390/ijms27167178)
Supplement: Supplementary file 1 [file ijms-27-07178-s001.zip › Supplementary Table S3.pdf]

Supplementary Table S3. Genes included within the KEGG "Leishmaniasis" pathway that are upregulated in mouse hepatic organoids co-cultured with *L- donovani*-mCh-infected macrophages in comparison with mouse hepatic organoids co-cultured with *L. donovani*-iRFP.

Gene Abbreviation	Gene Name[1]
C3	Complement component 3
Cyba	Cytochrome b-245, alpha polypeptide
Cybb	Cytochrome b-245, beta polypeptide
Elk1	ETS transcription factor ELK1.
Fcgr1	Fc receptor, IgG, high affinity I
Fcgr3	Fc receptor, IgG, low affinity III
Fcgr4	Fc receptor, IgG, low affinity IV
Fos	Fos proto-oncogene, AP-1 transcription factor subunit
H2-Aa	Histocompatibility 2, class II antigen A, alpha
H2-Ab1	Histocompatibility 2, class II antigen A, beta 1
H2-DMa	Histocompatibility 2, class II, locus DMa
H2-DMb1	Histocompatibility 2, class II, locus Mb1
H2-Ea	Histocompatibility 2, class II antigen E alpha
H2-Eb1	Histocompatibility 2, class II antigen E beta
Ifngr2	Interferon gamma receptor 2
Il10	Interleukin 10
Il1a	Interleukin 1 alpha
Il1b	Interleukin 1 beta
Itga4	Integrin alpha 4
Itgam	Integrin alpha M
Itgb2	Integrin beta 2
Jak1	Janus kinase 1
Jak2	Janus kinase 2
Jun	Jun proto-oncogene, AP-1 transcription factor subunit
Marcksl1	MARCKS-like 1
Ncf1	Neutrophil cytosolic factor 1
Ncf2	Neutrophil cytosolic factor 2
Ncf4	Neutrophil cytosolic factor 4
Nfkbia	Nuclear factor of kappa light polypeptide gene enhancer in B cells inhibitor, alpha
Nfkbib	Nuclear factor of kappa light polypeptide gene enhancer in B cells inhibitor, beta
Nos2	Nitric oxide synthase 2, inducible
Prkcb	Protein kinase C, beta
Ptgs2	Prostaglandin-endoperoxide synthase 2
Ptpn6	Protein tyrosine phosphatase, non-receptor type 6
Stat1	Signal transducer and activator of transcription 1(Stat1)
Tab2	TGF-beta activated kinase 1/MAP3K7 binding protein 2
Tgfb1	Transforming growth factor, beta 1
Tlr2	Toll-like receptor 2
Tnf	Tumor necrosis factor

[1]Information retrieved from the National Library of Medicine (https://www.ncbi.nlm.nih.gov/datasets/gene/).
