# Supplementary material for: An Immunocompetent Hepatic Organoid Model Reveals Conserved Hepatic Immune Dysregulation During Leishmania donovani Infection
Source: Int J Mol Sci. 2026 Aug 11;27(16):7178. doi: 10.3390/ijms27167178 (PMC13513185; doi:10.3390/ijms27167178)
Supplement: Supplementary file 1 [file ijms-27-07178-s001.zip › Supplementary Table S4.pdf]

**Supplementary Table S4.** Upregulated genes encoding proteins related to chemokine signaling and cytokines in *L. donovani*-infected mouse liver.

| Functional group | Gene name | Description |
|---|---|---|
| Chemokine System | *Ccl19, Ccl2, Ccl24, Ccl4, Ccl5, Ccl6, Ccl8, Cxcl1, Cxcl10, Cxcl13, Cxcl14, Cxcl16, Cxcl5, Cxcl9, Cxcl11, Pf4, Ppbp, Ccr2, Ccr5, Cxcr3, Cxcr4, Cxcr6, Xcr1* | Chemokines and their receptors involved in immune cell recruitment and migration |
| Cytokines / Inflammatory Mediators | *Il1b, Il18, Il33, Tnf, Tnfsf10, Tnfsf13b, Gdf3, Nampt, Ltb* | Secreted proteins that regulate immune activation, inflammation, and cell signaling |
| Intracellular Signaling Proteins | *Fgr, Grk3, Itk, Rac2, Src, Was, Adcy1, Adcy7, Dock2, Elmo1, Gnb4, Gng2, Gngt2, Hck, Ncf1, Pak1, Prex1, Pik3cd, Pik3cg, Pik3r5, Plcb2, Plcg2, Prkcb, Stat1, Vav1, Cmtm7* | Cytoplasmic kinases, adaptors, GTPases, and transcription factors involved in signal transduction |
