# Supplementary material for: An Immunocompetent Hepatic Organoid Model Reveals Conserved Hepatic Immune Dysregulation During Leishmania donovani Infection
Source: Int J Mol Sci. 2026 Aug 11;27(16):7178. doi: 10.3390/ijms27167178 (PMC13513185; doi:10.3390/ijms27167178)
Supplement: Supplementary file 1 [file ijms-27-07178-s001.zip › Supplementary Table S5.pdf]

Supplementary Table S5. Genes included within the KEGG "Leishmaniasis" pathway that are upregulated in *L. donovani*-infected mouse liver.

**Gene Abbreviation**	**Gene Name**[1]
Cyba	Cytochrome b-245, alpha polypeptide
Cybb	Cytochrome b-245, beta polypeptide
Fcgr1	Fc receptor, IgG, high affinity I
Fcgr3	Fc receptor, IgG, low affinity III
Fcgr4	Fc receptor, IgG, low affinity IV
H2-Aa	Histocompatibility 2, class II antigen A, alpha
H2-Ab1	Histocompatibility 2, class II antigen A, beta 1
H2-DMa	Histocompatibility 2, class II, locus DMa
H2-DMb1	Histocompatibility 2, class II, locus Mb1
H2-DMb2	Histocompatibility 2, class II, locus Mb2
H2-Ea	Histocompatibility 2, class II antigen E alpha
H2-Eb1	Histocompatibility 2, class II antigen E beta
H2-Ob	Histocompatibility 2, O region beta locus
Il1b	Interleukin 1 beta
Itga4	Integrin alpha 4
Itgam	Integrin alpha M
Itgb2	Integrin beta 2
Ncf1	Neutrophil cytosolic factor 1
Ncf2	Neutrophil cytosolic factor 2
Ncf4	Neutrophil cytosolic factor 4
Nos2	Nitric oxide synthase 2, inducible
Prkcb	Protein kinase C, beta
Ptpn6	Protein tyrosine phosphatase, non-receptor type 6
Stat1	Signal transducer and activator of transcription 1(Stat1)
Tlr2	Toll-like receptor 2
Tlr4	Toll-like receptor 4
Tnf	Tumor necrosis factor

[1]Information retrieved from the National Library of Medicine (https://www.ncbi.nlm.nih.gov/datasets/gene/).
